# Self-Assembly of Alkylamido Isophthalic Acids toward the Design of a Supergelator: Phase-Selective Gelation and Dye Adsorption

**DOI:** 10.3390/gels8050285

**Published:** 2022-05-05

**Authors:** Darren A. Makeiff, Jae-Young Cho, Bradley Smith, Rina Carlini, Nicolas Godbert

**Affiliations:** 1 Nanotechnology Research Center, National Research Council of Canada, 11421 Saskatchewan Drive, Edmonton, AB T6G 2M9, Canada; jae-young.cho@nrc.ca (J.-Y.C.); bradley.smith@nrc.ca (B.S.); 2 Optimal Innovation Group Inc., 175 Longwood Rd. S, Suite 112 A, Hamilton, ON L8P 0A1, Canada; rina@optimalinnovationgroup.com; 3 Dipartimento di Chimica e Tecnologie Chimiche, Università della Calabria, 87036 Rende, Italy; nicolas.godbert@unical.it

**Keywords:** supramolecular organogel, self-assembly, isophthalic acid, Hansen solubility parameters, rheology, phase-selective gelation, dye adsorption

## Abstract

A new series of 5-alkylamido isophthalic acid (ISA) derivatives with varying single and twin alkyl chain lengths were designed and synthesized as potential supramolecular organogelators. 5-alkylamido ISAs with linear or branched alkyl tail-groups of different lengths were effective gelators for low polarity solvents. In particular, among the presented series, a derivative with a branched, 24 carbon atom tail-group behaves as a “supergelator” with up to twenty organic solvents forming gels that are highly stable over time. The gelation behavior was analyzed using Hansen solubility parameters, and the thermal stability and viscoelastic properties of select gels were characterized. Microscopy, spectroscopy, powder X-ray diffraction, and computer modeling studies were consistent with a hierarchical self-assembly process involving the formation of cyclic H-bonded hexamers via the ISA carboxylic acid groups, which stack into elementary fibers stabilized by H-bonding of the amide linker groups and π–π stacking of the aromatic groups. These new nanomaterials exhibited potential for the phase-selective gelation of oil from oil–water mixtures and dye uptake from contaminated water. The work expands upon the design and synthesis of supramolecular self-assembled nanomaterials and their application in water purification/remediation.

## 1. Introduction

Supramolecular gels from low molecular weight gelators (LMWGs) [1,2,3,4,5,6,7,8,9] are a fascinating class of soft materials that have recently garnered significant interest in many biological and material applications [10,11,12]. These materials have shown great potential in areas such controlled release/drug delivery [13,14], tissue engineering [15], sensors [16], template materials [17], catalysis [18], cosmetics [19], foods [20], optics and electronics [21], and environmental remediation [22,23], as well as many others [4,10,11,12]. In most cases, LMWGs undergo hierarchical self-assembly to form one-dimensional (1D) fiber-like structures that eventually entangle into three-dimensional (3D), self-assembled fibrillar networks (SAFINs) [1,2,3,4,5,6,7,8,9,24]. Solvent molecules become confined within the pores of the SAFIN via capillary forces as the bulk liquid is rigidified into a viscoelastic solid-like gel [1,2,3,4,5,6,7,8,9]. Supramolecular gels from LMWGs are physical gels and thus form reversibly, driven by the formation of non-covalent interactions such as H-bonding, π–π stacking, van der Waals, and metal–ligand interactions [1,2,3,4,5,6,7,8,9,24]. Although a large number of LWMGs have been reported to date, the rational design of LWMGs for the predictable gelation of specific liquids to yield gels with specific properties still remains a significant challenge.

**Figure 1:**
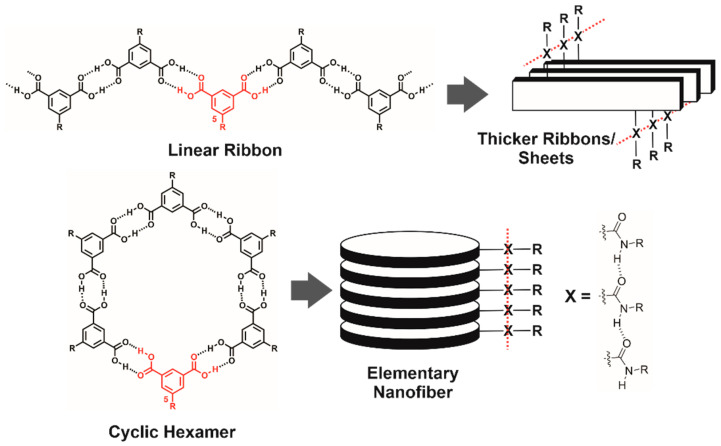
Hierarchical self-assembly of ISA derivatives.

The isophthalic acid (ISA) group (Figure 1) is a fascinating supramolecular synthon used in a variety of unique and programmable supramolecular architectures [25,26,27,28,29,30,31,32,33,34,35,36]. Many ISA derivatives self-associate to form either intermolecular, linear polymeric ribbons [37,38], or disk-shaped cyclic hexamers [38,39] via H-bonded carboxylic acid dimers (Figure 1). Supramolecular gelation of ISA derivatives has been accomplished via the incorporation of a secondary H-bonding group (i.e., amide or urea) to drive the formation of fibrous structures either via stacking of (1) H-bonded ribbons into thicker ribbons or sheets or (2) cyclic hexamers (Figure 1) [40]. However, only two reported ISA gelators self-assemble via these pathways (Figure 1) [40,41], while most ISA-based supramolecular gelator systems involve different H-bonding motifs and/or driving forces [42,43,44,45,46,47,48,49,50,51,52,53,54].

Recently, supramolecular gels have received significant attention for water remediation applications including oil spill clean-up and adsorbents for the removal of contaminants from industrial wastewater [22,23,55]. The phase-selective gelator behavior of LMWGs for oils from oil–water mixtures may be useful to clean up marine oil spills, where the solidified oil phase can be mechanically separated from water more easily than the fluid form [56]. Subsequently, both the oil and LMWG can be easily recovered after distillation [57]. Moreover, the high surface area and porosity of gels and the ability of the constituent LMWGs to form non-covalent interactions with neutral, anionic, or cationic contaminants has been demonstrated to be effective molecular adsorption from either wet or dried hydrogel or organogel phases [23,58,59,60]. As a result, supramolecular gels have demonstrated great potential as solid phase adsorbents for the removal of toxic contaminants or to selectively remove components from complex aqueous mixtures to simplify analysis [61]. However, despite these advances, significant challenges such as long adsorption time and low adsorption capacity need to be improved before these materials can be put practice on large scales in real situations.

**Figure 2:**
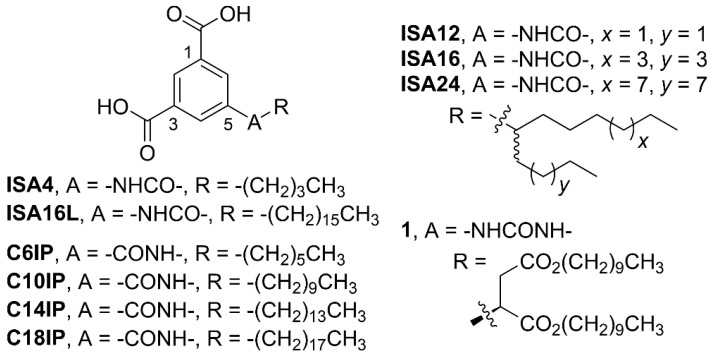
5-alkylamido isophthalic acid derivatives.

Here, we report the design and synthesis of five new alkylated ISA derivatives (Figure 2) and their supramolecular self-assembly and gelator behavior. These five compounds and other related derivatives and the potential application of new materials derived from these compounds have already been disclosed previously in several patents [62,63,64,65,66]. The compounds reported here differ from other related, reported ISA gelators [40,41,42] by the structure of the alkyl tail-groups and the type and direction of the secondary H-bonding linker group. The gelation behavior of these compounds in a variety of organic solvents was analyzed using Hansen solubility parameters (HSPs), and the gel properties and self-assembly driving forces were examined. Finally, the application of these compounds towards the removal of unwanted pollutants from contaminated water is also discussed.

## 2. Results and Discussion

### 2.1. Synthesis

We recently reported a series of 5-alkylamido benzimidazolone (BZI) supramolecular gelators [67]. BZI compounds with single alkyl tail-groups were ineffective gelators, while branched, twin, Guerbet-type tail-groups were effective gelators for many different organic solvents [67]. A similar design was used for this work, where the BZI head-group is replaced with ISA. The chemical structures of the 5-alkylated ISA derivatives synthesized here are shown in Figure 2. All five compounds were characterized using ^1^H NMR, ^13^C NMR, and high-resolution mass spectrometry (see Figures S1–S15, Supporting Information).

The chemical structures of the 5-alkylamido ISA compounds in Figure 2 (i.e., **ISA4**, **ISA16L**, **ISA12**, **ISA16**, and **ISA24**) are different than the alkylated ISA gelators previously reported by Hamilton et al. [40] (compound **1**) or Lv et al. (compounds **C6IP**, **C6IP**, **C14IP** and **C18IP**) [41]. Both were proposed to self-assemble via H-bonded cyclic hexamer or linear ribbon motifs (Figure 1). Hamilton et al.’s 5-alkylurea ISA gelator **1** features a urea instead of an amide linker group, an additional aspartic acid moiety, and different hydrocarbon tail-groups (i.e., two decyl chains) [40]. Lv et al.’s alkanoyl ISAs have different, non-branched hydrocarbon tail-groups (i.e., hexyl, decyl, tetradecyl, and octadecyl chains), and the amide linker-group is reversed, compared to the compounds reported here (Figure 2) [41]. Most importantly, the synthesis of the compounds here (Scheme S1) was more efficient and without the need for tedious chromatography purification steps.

### 2.2. Gelation Behavior

The gelation behavior of all 5 alkylamido ISAs was tested in up to 37 organic solvents (Table S1). The compound **ISA24** possessed the bulkiest tail-group, was the easiest to solubilize in nearly all 37 solvents, and formed gels with 30 different solvents. Although **ISA12**, **ISA16**, and **ISA16L** were significantly more difficult to completely solubilize, gels were formed with thirteen, eleven, and thirteen different solvents, respectively (Table S1). The four 5-alkylamides **ISA12**, **ISA16**, **ISA16L**, and **ISA24** were best suited for gelling low polarity, aprotic solvents (i.e., aromatics, halocarbons, linear and cyclic aliphatic hydrocarbons), although **ISA16**, **ISA16L**, and **ISA24** all gelled the highly polar solvent ethylene glycol at low gelator concentrations. Most gels that formed with polar solvents (i.e., ethanol, 1-hexanol, and acetone) required considerably higher gelator concentrations (i.e., >2 wt%, Table S1). The gelators **ISA12**, **ISA16**, **ISA16L**, and **ISA24** were also “supergelators” for at least nine, six, eight, and twenty different solvents, respectively, at concentrations of 1 wt% or lower. The term “supergelator” has been commonly used throughout the literature for several decades in the field of supramolecular gels. In a general manner, this term is used to qualify LMWGs that are able to gel a large amount of solvents at low concentration, from 1% *w*/*w* and lower. To the best of our knowledge, the term was first introduced in 1994 by Shinkai et al. to describe the gelation behavior of some cholesterol derivatives [68], and the lowest gelator concentrations reached so far (0.03–0.07%) were reported for α-D-galactopyranoside and α-D-mannopyranoside derivatives [69]. 

Clearly, the best overall gelator was **ISA24**, due to versatility in gelling the most different types of solvents and efficiency in forming gels at lower gelator concentrations. Indeed, the gels from **ISA12**, **ISA16**, and **ISA16L** were more challenging to form, requiring higher temperatures and longer heating times, due to the significant decrease in viscosity, which also inhibited proper mixing. Furthermore, many gels that did form from **ISA12**, **ISA16**, and **ISA16L** were unstable over time, either collapsing to partial gels or releasing significant amounts of solvent via syneresis (i.e., decalin and aromatic solvents, Table S1). The compound with the shortest tail-group, **ISA4**, was the only compound that did not give any positive inversion test results. Poor solute–solvent interactions and favorable packing interactions amongst the short butyl tail-groups of **ISA4** likely lead to more highly ordered self-assembled structures that result in precipitates or crystallites, rather than providing the steric stabilization required to form sample spanning, self-assembled fibrillar networks (SAFINs) and gels.

Note that the chemical structure of the gelator **ISA16L** is similar to the previously reported gelators **CP14IP** and **CP18IP** (Figure 2) [41]. The only differences are the reversed direction of the amide linker group and minus or plus two methylene groups, respectively (Figure 2). Although no data were reported on the gelation behavior of **CP14IP** and **CP18IP** with low polarity solvents, both compounds were good gelators for high polarity ethanol:water (97:3 to 92.5:7.5) solvent mixtures at 10 mM [41]. For this work, **ISA16L** only formed crystallites/precipitates and not gels with similar ethanol:water solvent compositions at 10 mM. Clearly, the subtle structural change of reversing the direction of the amide group has a significant impact on the ability of the 5-alkylamido ISA compounds to form gels with polar solvents. 

### 2.3. Hansen Solubility Parameters

HSPs were employed to rationalize the outcome of the gelation experiments. HSPs are a powerful empirical tool that were initially developed to select solvents for polymers, which have been more recently applied to predict the gelation behavior of LMWGs [70,71,72,73,74]. Other approaches involving solvent parameters and computational approaches have been developed, however, HSPs are perhaps regarded as the best approach, as HSPs consider multiple parameters and are relatively simple to use [71,74]. The HSP approach takes into account three types of intermolecular interactions, i.e., dispersive (δ_d_), polar (δ_p_), and H-bonding interactions (δ_h_) in MPa^1/2^ [70]. Typically, the outcomes for gel tests with common solvents and LMWGs are categorized as gel (G), soluble (S), and insoluble (I) for suspensions and precipitates, and the results are plotted in 3D “Hansen Space” [70,71,72,73,74]. Cluster analysis is then carried out to fit the gel regions with spheres with HSP values, i.e., origin coordinates and a radius size [70,71,72,73,74].

The results of the gelation tests with different solvents (Table S2) at 1 wt% were plotted in 3D Hansen space using the HSPs for each solvent (Figure S16), which were obtained from the literature [75]. Significant overlap was observed amongst all four LMWG gel spheres, especially for the three smaller gelators **ISA12**, **ISA16**, and **ISA16L**. For the branched gelators **ISA12**, **ISA16**, and **ISA24**, the increase in tail-group size progressively shifted the gel sphere center to higher δ_p_ values (2.51–5.98 MPa^1/2^), higher δ_h_ values (2.51–5.98 MPa^1/2^), and lower δ_d_ values (19.72–18.20 MPa^1/2^) as the sphere radius increased (5.73–6.76 MPa^1/2^, Table S3). In other words, with increasing tail-group size, gelator–gelator polar and H-bonding interactions become stronger, while dispersive interactions weaken relative to similar gelator–solvent interactions. The increase in sphere radius indicates that the gelator versatility (i.e., the ability to gel more different types of solvents) increases with gelator tail-group size.

**Figure 3:**
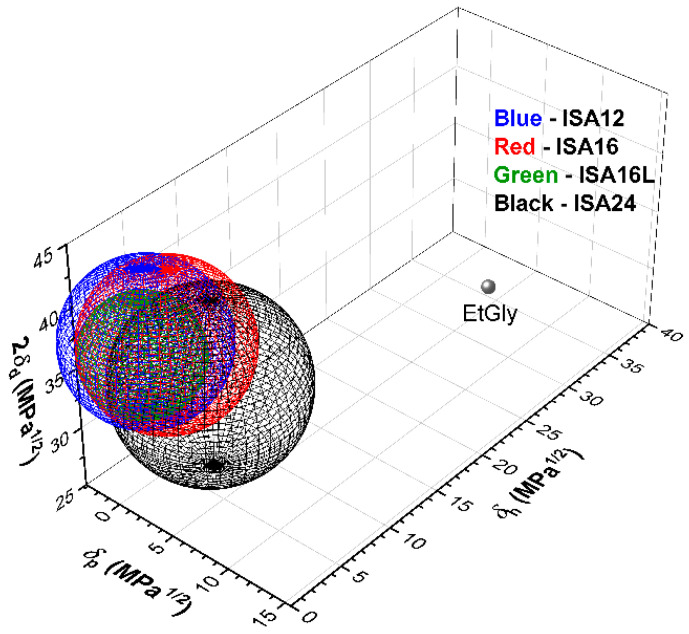
Hansen space for 5-alkylamido isophthalic acid gelators (1 wt%). EtGly = ethylene glycol.

Comparison of **ISA16L** and **ISA16** shows the effect of branching on gelation behavior. **ISA16L** and **ISA16** are constitutional isomers with a linear sixteen carbon, and twin ten and six carbon tail-groups, respectively. The effect of branching increased the sphere radius from 4.27 to 5.88 MPa^1/2^ (Table S3), which suggests that branched structures improve gelator–gelator interactions relative to solvent–gelator interactions. Note that the data point for ethylene glycol is shown as a single outlier in Figure 3 for **ISA16**, **ISA16L**, and **ISA24** and may indicate the presence of a second gelation region at high δ_p_ and δ_h_ values. The HSP results here could be used to predict the gelator properties of each gelator in other single or multicomponent solvent mixtures not tested here that would be useful for specific applications.

### 2.4. Thermal Stability

**Figure 4:**
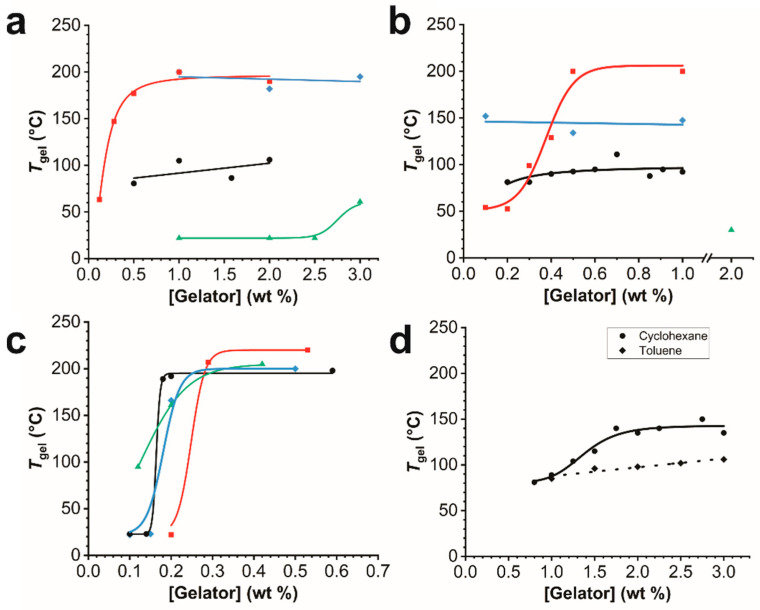
Gel-to-sol transition temperature (*T*_gel_) dependence on gelator concentration for gels with (**a**) xylenes, (**b**) decalin, (**c**) paraffin oil, and (**d**) cyclohexane (solid) and toluene (dotted). **ISA12** (diamonds, blue), **ISA16** (squares, red), **ISA16L** (triangles, green), and **ISA24** (circles, black).

To assess the thermal stability of the gels, the falling ball method was used to determine the gel-to-sol transition temperatures (*T*_gel_) at various gelator concentrations (Figure 4) [76]. Experiments were carried out on gels with xylenes, decalin, and paraffin oil to examine the effect of tail-group structure on *T*_gel_ with different types of hydrocarbon liquids (i.e., aromatics, cycloaliphatics, and linear, aliphatic hydrocarbon mixtures, respectively). For most gelator–solvent combinations, *T*_gel_ increased very sharply with gelator concentration over a narrow concentration range (i.e., 0.05–0.5 wt%) just slightly above the critical gelator concentration (CGC). In general, for the gels with xylenes and decalin, the maximum *T*_gel_ (*T*_gel_ (max)) increased with decreasing tail-group length. *T*_gel_ (max) increased in the order of **ISA16L** (50 °C) < **ISA24** (~90 °C) < **ISA12**~**ISA16** (~190 °C) for xylenes (Figure 4a) and **ISA16L** (23 °C) < **ISA24** (~90 °C) < **ISA12** (~150 °C) < **ISA16** (~200 °C) for decalin (Figure 4b), while tail-group length decreased in the order of **ISA16L** < **ISA24** < **ISA16** < **ISA12**. Longer hydrocarbon tail-groups are more hydrophobic and probably promote more favorable gelator–solvent interactions and weaker gels with low polarity solvents, while shorter, more hydrophilic tail-groups promote more favorable gelator–gelator interactions and more robust gels with low polarity solvents. Note that the gels of **ISA16L** with xylenes and decalin were weak and required high gelator concentrations (1–2 wt%) to just support the ball at 23 °C, despite the relatively low CGC values (<0.4 wt%, Table S1).

In contrast, *T*_gel_ (max) for the paraffin gels with each gelator were very similar (~200–220 °C). However, the minimum concentration at which *T*_gel_ (max) occurs decreased in the order of **ISA24** (0.15 wt%) < **ISA16L**~**ISA12** (~0.25 wt%) < **ISA16** (~0.30 wt%), which correlates well with decreasing tail-group length, despite similar CGCs (0.1–0.2 wt%, Table S1). Paraffin oil typically consists of saturated hydrocarbons between 5–15 carbon atoms in length, which may enable a balance of gelator–gelator and gelator–solvent interactions for a range of different gelator tail-group structures. Interestingly, the gels from **ISA12** and **ISA16** with xylenes, and gels from **ISA16** with decalin, exhibited exceptional thermal stability, with *T*_gel_ (max) values 10–60 °C greater than the solvent bps (i.e., ~138 and ~190 °C, respectively). Although **ISA24** was the best overall gelator, the gels with xylenes and decalin did not exhibit the highest thermal stability (Figure 4a,b, respectively).

Figure 4d also compares the effect of gelator concentration on *T*_gel_ for gels from **ISA24** with cyclohexane and toluene. For cyclohexane, *T*_gel_ exhibited a linear increase between the CGC (~0.7 wt%) and the minimum concentration at which *T*_gel_ (max) of ~140 °C occurs (~1.8 wt%). In contrast, for toluene, *T*_gel_ only increased moderately from 85 °C to 106 °C over the same concentration range, similar to the gels of **ISA24** with xylenes (Figure 4a). The high *T*_gel_ (max) for cyclohexane is intriguing, considering that *T*_gel_ (max) is 60 °C higher than the bp of neat cyclohexane (~80 °C), and that the bp of cyclohexane is considerably less than neat toluene (~110 °C). Above 2 wt%, no apparent change in gel consistency was observed. Further heating above *T*_gel_ (max) caused violent solvent evaporation.

Note that for most experiments, as the ball passed throughout the gel, no noticeable change in appearance to a liquid phase was observed. This observation is consistent with *T*_gel_ values occurring well above the bp of the liquids, and consequently partial decomposition of the gels (i.e., gel decomposition temperature, *T*_d_) must occur at this temperature rather than true gel-to-sol transitions [77]. This high thermal stability can be correlated to the self-assembly process in the gel state that is probably governed by strong intermolecular interactions such as H-bonds.

### 2.5. Rheological Measurements

The mechanical properties of materials are important for practical applications, and for organogels are deeply correlated to their viscoelastic behaviors. For this reason, we carried out rheological measurements on the best performer of the presented series, **ISA24**. The choice to reduce the rheological investigation only to **ISA24** was dictated by considering two factors: (1) the series studied is highly homogeneous in chemical structure since all compounds only differ from the length or type of the alkyl chains (linear vs. branched), (2) only **ISA24** formed gels that were suitably stable upon scale-up for rheological examination. Gels from **ISA12**, **ISA16**, and **ISA16L** with low polarity solvents (i.e., xylenes and decalin) were weak and unfortunately unstable over time. The alkyl chains may be either too short (i.e., **ISA12** and **ISA16**) to provide effective steric stabilization and/or favorable tail-group–solvent interactions over a long period of time, or are long enough (i.e., **ISA16L**), but may be prone to favorable inter-fiber tail-group stacking interactions due to the linear structure, which eventually leads to phase separation and precipitation over time.

Organogels of **ISA24** with decalin, toluene, cyclohexane, and crude oil were examined at 1 wt%. For xylenes, 2 wt% gels were used, since stable gel samples at larger scales suitable for rheological measurements could not be produced at 1 wt%. True gels exhibiting solid-like behavior in the linear viscoelastic region (LVR) are characterized by a storage/elastic modulus (*G′*) that is several times greater than the loss/viscous modulus (*G″*), and that *G′* and *G″* are independent of frequency [78,79]. Typically, strain sweep experiments are first carried out to determine the LVR and suitable strain values for frequency sweep experiments. More specifically, the strain limit is above which the gel networks begin to break down [60,61]. *G′* deviates from linearity above these strain values until the yield or crossover point is reached, where *G′* and *G″* crossover takes places, i.e., *G′* = *G″*, the gel-to-sol transition.

**Figure 5:**
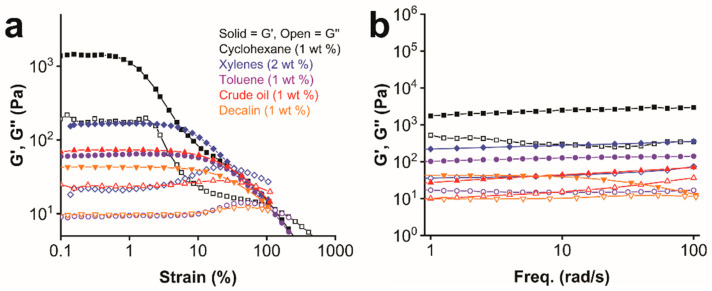
Viscoelastic response of the organogels of **ISA24** with different organic liquids. (**a**) Strain sweep. (**b**) Frequency sweep.

The dependence of the strain limit and the crossover strain on the solvent mostly followed similar trends (Figure 5a). The strain limit increased in the order of cyclohexane (0.7%) < xylenes (3.1%)~decalin (3.2%) < crude oil (4.0%) < toluene (5.3%), while the crossover strain increased in the order of xylenes (34%) < decalin (50%) < crude oil (60%) < toluene (108%) < cyclohexane (134%). Interestingly, the cyclohexane gel was the exception, whose gel network started to break down at lower strain values, despite exhibiting higher resilience to complete breakdown than the gels with the other solvents. Aromatic solvents such as toluene and xylenes and crude oil consist of aromatic hydrocarbons and polyaromatic hydrocarbons (i.e., asphaltenes), which may weaken the gel networks from **ISA24** by interfering with π–π stacking interactions. Overall, all of these gels have relatively large tan*δ* (*G″*/*G′* > 0.1) values, which are typical of so-called weak gels [80].

Frequency sweeps of the organogels (Figure 5b) revealed that *G′* was independent of frequency over the measured range and 4–8 times higher than *G″* over the entire frequency range. Although *G′* and *G″* data here were collected using a conventional rheometer and are typical of a viscoelastic material such as a gel, others have shown that for low frequencies and long relaxation times this may not be the case [8,81]. At low frequencies, supramolecular gels from LMWGs may experience irreversible permanent deformations and may not actually be physical gels, but rather may be “solid-like networks that respond elastically for small deformations and are embedded in suspending fluids [8,81].” For **ISA24** in this work, the elastic modulus, *G*′, which is a measure of gel stiffness, increased in the order of decalin (40 Pa) < toluene (60 Pa) < crude oil (70 Pa) < xylenes (170 Pa) < cyclohexane (1380 Pa). Therefore, gel stiffness correlates well with the relative gel strengths, which is not always the case for all gels. The rheology data show the relative stability or elasticity of the gels under mechanical stress, which likely reflects differences in the nano and microstructures of the gel networks formed in the different liquids and is important for most applications involving manipulation and transportation.

### 2.6. Microscopy Studies

Self-assembled nanostructures from all five 5-alkylamido ISA derivatives were imaged by scanning electron microscopy (SEM). All five compounds formed ultrafine, self-assembled nanofibers with widths <10 nm (Figure S17). Individual nanofibers best visualized in images prepared by depositing and drying 0.1–1 mg/mL solutions (Figures S17–S26). Ultrafine nanofibers of **ISA4** were observed from xylenes:THF (1:1), even though **ISA4** did not form any gels (Figure S18), while nanofibers of **ISA12**, **ISA16**, and **ISA16L** were observed from toluene, xylenes, and xylenes:THF mixtures (Figures S17, S19 and S20). THF was added as a co-solvent to improve solubility. Ultrafine nanofibers of **ISA24** were observed from cyclohexane (Figure S21), toluene (Figure S22), and chloroform (Figure S23), as well as THF (Figure S24).

**Figure 6:**
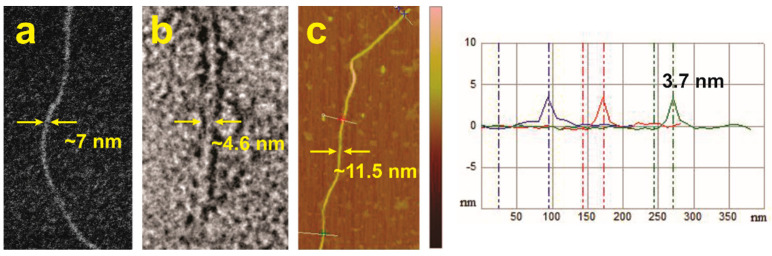
Microscopy images of elementary nanofibrils of **ISA24** deposited from cyclohexane solution (0.1 wt%). (**a**) Scanning electron microscopy (SEM) image. (**b**) High–resolution transmission electron microscopy (HR–TEM) image. The contrast enhanced with a negative uranyl acetate stain. (**c**) Atomic force microscopy (AFM) height image (1.955 μm × 1.065 μm, vertical scale bar represents 0–10 nm) with a cross-sectional analysis across an individual elementary nanofiber in three different places.

Figure 6 shows representative images of the smallest nanofibers of **ISA24**, which are probably elementary nanofibers, based on their dimensions from SEM (Figure 6a), high-resolution transmission electron microscopy (HR-TEM, Figure 6b and Figure S25), and atomic force microscopy (AFM, Figure 6c and Figure S26) measurements. The width of the nanofibers were measured to be ~7.0, ~4.6, and ~11.5 nm, respectively. The value of ~4.6 nm is considered to the most accurate, since HR-TEM images are direct 2D projections of the self-assembled nanostructures. SEM and AFM widths of soft materials can be artificially large due to edge electron scattering effects and tip convolution broadening effects [82], respectively. AFM cross-section height measurements (Figure 6c and Figure S26) gave an average height of ~3.6 nm for individual nanofibrils and relatively small, flat bundles (2–6 elementary nanofibers wide), which is slightly lower than the HR-TEM width. However, these values are in fairly good agreement considering that the AFM height may be underestimated due to the known limitations of AFM towards flattening soft matter nanostructures [79].

These results suggest that the elementary nanofibers have symmetrical cross-sections, which is consistent with the expected stacks of cyclic, H-bonded hexamers based on the widths of computer models of the cyclic hexamers (Table S4 and Figure S27). The experimentally measured diameter of 3.6–4.6 nm is in good agreement with the dimensions determined from computer models of cyclic hexamers from **ISA24**. A cyclic hexamer of **ISA24** (Figure 1) is expected to have a diameter between 2.3 to 5.2 nm, where the minimum of 2.3 nm accounts for only the rigid core and the maximum of 5.2 nm represents the rigid core with fully extended alkyl chains. In contrast, the cross-sections of linear ribbons (Figure 1) are expected to be only a few atoms thick (i.e., <0.5 nm), whereas stacks of cyclic hexamers are several molecules thick (>0.5 nm) [83]. Furthermore, nanofibers from stacks of ribbons would be expected to give less uniform cross-sections than observed, owing to a statistical distribution of heights/widths, regardless of the orientation relative to the substrate. 

**Figure 7:**
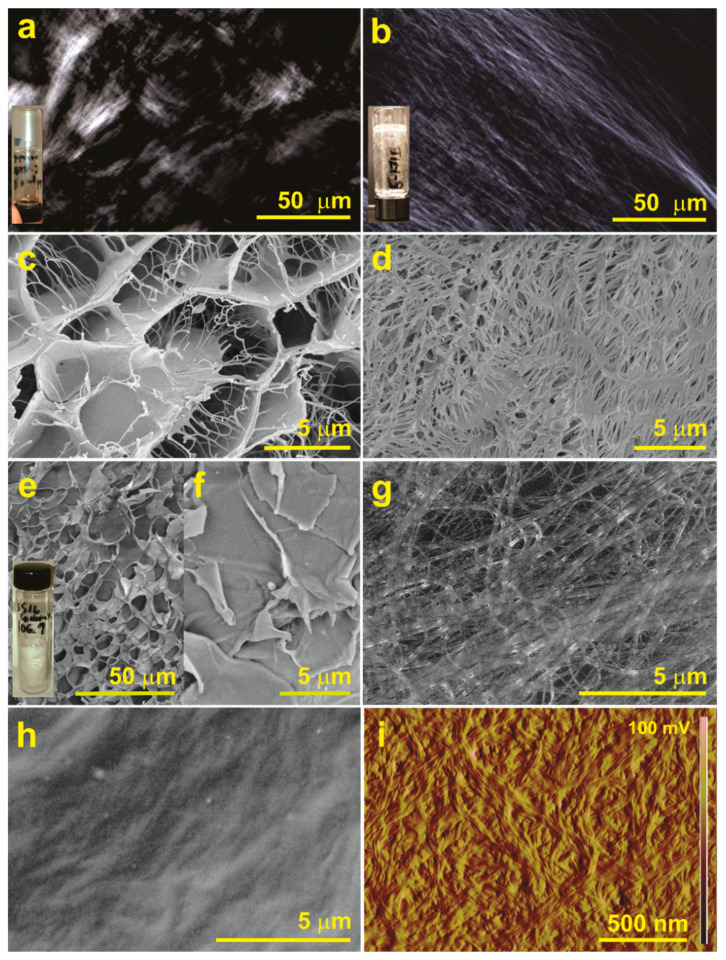
Microscopy images of SAFINs of **ISA24**. POM images of organogels of **ISA24** with (**a**) cyclohexane and (**b**) toluene (2 wt%). Freeze-fractured cryo-SEM images of in situ aerogels of **ISA24** from (**c**) cyclohexane and (**d**) toluene organogels (2 wt%). SEM images of an ex situ, freeze-dried aerogel of ISA24 from a cyclohexane organogel (2 wt%) at 23 °C (**e**,**f**). SEM images of dense, in situ xerogels of **ISA24** from (**g**) toluene and (**h**) cyclohexane (2 wt%) at 23 °C. AFM amplitude images of a thick in situ xerogel of ISA24 from cyclohexane (2 wt%) (**i**).

Polarized optical microscopy (POM) images of organogels from **ISA24** exhibited strong birefringence (Figure 7a,b and Figure S28), which is expected for highly ordered, anisotropic structures. While the presence of nanofibers of **ISA24** was inconclusive for the cyclohexane gel (Figure 7a), nanofibers were clearly present for the toluene gel (Figure 7b). Consistent with these results, cryo-SEM images of in situ aerogels formed upon removal of the corresponding solvents clearly show different SAFIN morphologies (Figure 7c,d). In the context of this report, in situ indicates that the aerogel or xerogel was formed at small scale (i.e., <0.1 mL) directly on the substrate for cryo-SEM analysis, while ex situ refers to aerogel samples that were prepared separately on a larger scale (i.e., 1–20 mL), and deposited onto a substrate for room temperature SEM analysis (see experimental section). In addition, the aerogels described here correspond to samples that were freeze-dried, while xerogels correspond to samples that were not frozen before solvent removal. The in situ aerogel from cyclohexane exhibited two phases (Figure 7c) consisting of honeycomb structures and sparse networks of nanofibers with widths between 40–230 nm, spanning the honeycomb pores, whose diameters range between a few μm to tens of μm. In contrast, the toluene sample (Figure 7d) was a single phase consisting of a dense network of significantly larger nanofibers with widths between 90–1400 nm and pores with diameters from tens of nm to one μm. The results strongly suggest that solvent type has a significant effect on the microstructure of the SAFINs formed from **ISA24** nanofibers.

An ex situ aerogel was also prepared at 10 mL scale, which resulted in a brittle monolith that retained most of the volume of the initial organogel. SEM images of the ex situ aerogel also exhibited honeycomb structures (Figure 7e). The majority of the sample consisted of flakes with smooth surfaces (Figure 7f). The low yield of the honeycomb structures is likely due to crushing the sample to flakes to facilitate removal from the sample vial mold. Ex situ aerogels from toluene were not prepared due to the low melting point of toluene (i.e., −95 °C), which is not conducive for freeze-drying using common commercial freeze-driers designed for water. Only solvents such as cyclohexane or benzene have melting and boiling points (i.e., ~5 °C and ~80 °C, respectively) close to water (0 °C and 100 °C, respectively).

The difference in the morphology of the SAFINs of **ISA24** formed from cyclohexane versus toluene are likely due to the large difference between the freezing points (fps) of cyclohexane and toluene (6.5 and −95 °C, respectively). The honeycomb structure observed from cyclohexane is due to the formation of solvent crystals, which can act as porogens, by causing the self-assembled nanofibers of **ISA24** to become concentrated and squeezed onto the crystallite boundaries to form membrane structures [84,85]. This effect is known to occur with solvents having high melting temperatures such as cyclohexane, water, and others [84,85]. The presence of nanofibers in the in situ aerogel, which are absent in the ex situ aerogel, probably results from the faster freezing rate of the significantly smaller sample size (i.e., μL versus several mL). Rapid freezing of a smaller sample may enable “trapping” of individual nanofibers before they are pushed to the solvent crystal boundaries. Therefore, we speculate that the native gel structure formed with cyclohexane more accurately resembles the SAFIN formed from toluene, except the average nanofiber widths are significantly smaller. In fact, cryo-SEM images did show several regions that agree with that hypothesis (i.e., Figure S29d, red arrow). In addition, the surface of the membrane structures forming the honeycomb walls likely resemble the collapsed xerogel films shown in Figure 7h,i.

Xerogels are most commonly analyzed by microscopy under the assumption that the network structures are related to those in the native, wet organogel state [86]. Here, the morphology of xerogels of **ISA24** cast from dried films of gels with toluene, cyclohexane, and other solvents are shown in Figure 7g–i and Figure S30, respectively. While the morphology of the xerogel from toluene was remarkably similar to the corresponding in situ aerogel, the size of the nanofibers was smaller (30–600 nm). In contrast, the xerogel from cyclohexane (Figure 7h,i) consisted of a dense network of ultrafine nanofibers (Figure 7i), which although they are too small to be resolved in SEM images (Figure 7h), could be easily seen at the gel edges (Figure S21a) as well as easily resolved by AFM (Figure 7i). The AFM widths of the nanofibers in Figure 7i are consistent with the AFM width of the elementary nanofiber in Figure 6c (i.e., ~10 nm).

The results in Figure 6 and Figure 7 suggest a relationship between SAFIN nano/microstructure and mechanical properties for the cyclohexane versus toluene gel. For some gels, bundling leads to thicker nanofibers that should have better mechanical properties [67,87], although this is not entirely true for the present work, i.e., smaller nanofibers are observed in cyclohexane than toluene, and the gel with cyclohexane is overall both stronger and stiffer. The lower upper strain limit for the LVR for the gel with cyclohexane (~0.7%) is significantly lower than toluene (~5.3%), which is consistent with the initial breakage of the smaller, mechanically weaker bundles observed in the SAFINs from cyclohexane (40–230 nm) instead of toluene (90–1400 nm). However, the higher stiffness and overall mechanical strength of the gels with cyclohexane (~1380 Pa and 134%, respectively) instead of toluene (~60 Pa and 108%, respectively) is consistent with the organization of smaller nanofibers into larger, more robust structures, such as the thick sheets defining the honeycomb network shown in Figure 7c,e, or a network of much smaller nanofiber bundles, which form a dense, more highly cross-linked SAFIN as shown in Figure 7i and Figure S29d (red arrow). The known phenomenon of the formation of honeycombs due to solvent freezing effects strongly suggests that the latter best represents the native structure for the gel with cyclohexane.

Since many electron microscopy techniques require high vacuum conditions, and often powder X-ray diffraction patterns are dominated by the amorphous nature of the solvent (the major component of the gel), gel samples are most commonly examined as “xerogels”, which are the SAFIN gel networks that result after the solvent has been removed. However, during the solvent evaporation process, capillary forces are known to eventually induce disruptions and collapse of the native gel network [86]. Therefore, the dried gels (xerogels and/or aerogels) are often assumed to have similar morphology as the native gel network, although it may not always be ensured. In any case, the different morphologies observed from different solvents (Figure 7, Figures S18–S24 and Figures S28–S30) strongly suggest the solvent molecular properties play a key role in the resulting SAFIN morphology.

### 2.7. Nuclear Magnetic Resonance (NMR) Spectroscopy

^1^H NMR studies were carried out to probe intermolecular interactions involved in the self-assembly of **ISA24** in CDCl_3_. Interestingly, at concentrations precluding gel formation (i.e., 1.9 mM), the signals for **ISA24** were absent (Figure S31). In contrast, at the same concentration in DMSO-*d*_6_, the signals for **ISA24** are sharp and strong (Figure S31c). **ISA24** does not gel DMSO-*d*_6_, and probably exists as free molecules since DMSO-*d*_6_ is a well-known strong H-bond acceptor that disrupts many H-bonded assemblies. The absence of signal in CDCl_3_ is consistent with the complete absence of any detectable mobile, free gelator, or low-order aggregates, because they are all incorporated into large, polymeric, low mobility self-assembled aggregates (i.e., nanofibers and/or SAFINs), which are usually not visible by solution-state ^1^H NMR spectroscopy due to the strong signal broadening induced by long correlation times [88,89,90,91].

### 2.8. ATR-FTIR Spectroscopy

Attenuated reflectance Fourier transform infrared spectroscopy (ATR-FTIR) was carried out to gain further insight into the self-assembly of **ISA24** in the bulk powder, organogel, and xerogel states. The stretching frequency of carbonyl groups of H-bonded dimers is well known to decrease relative to other, more weakly H-bonded or non-H-bonded (monomeric) forms. ATR-FTIR spectra of **ISA24** in all three states showed broad carbonyl bands centered between 1693–1699 cm^−1^ and ~1660 cm^−1^, which are consistent with H-bonded carboxylic acid dimers and H-bonded amide carbonyl groups, respectively (Figure S32) [30,92,93,94].

The position of the carbonyl bands for **ISA24** are consistent with FTIR data for other ISA derivatives that form cyclic hexamers. Recently, Sockalingam et al. reported a carbonyl stretch at ~1695 cm^−1^ for a cyclic H-bonded hexamer of 5-hydroxyISA with an 18-crown-6 and seven water molecules in the cavity [93], while Zimmerman et al. reported carbonyl stretches at ~1712 and 1690 cm^−1^ for carboxyl acid dimers of double, cyclic H-bonded ISA hexamers functionalized with dendritic groups [92]. In this work, an additional carbonyl band was observed at ~1718 cm^−1^ in the ATR-FTIR spectrum for the bulk powder (Figure S32), which probably corresponds to a weakly H-bonded or non-H-bonded carbonyl group (i.e., **ISA24** monomer).

In the amide II region, the ATR-FTIR spectra of the bulk powder, xerogel, and organogel states display vibrational bands at 1539, 1549, and 1556 cm^−1^, respectively, which are due to N-H bending of the amide linker groups (Figure S32). The position of these signals is characteristic of H-bonded amide N-H groups. The increased blue-shift from the bulk powder to the xerogel to the gel indicates the amide N-Hs are involved in increased H-bonding interactions in that order. These results all clearly confirm (1) the formation of H-bonded carboxylic acid dimers in all three states, which is consistent with cyclic, H-bonded hexamers, and (2) H-bonded amide groups, which is consistent with stacks of cyclic hexamers stabilized by H-bonds.

### 2.9. Powder X-ray Diffraction

**Figure 8:**
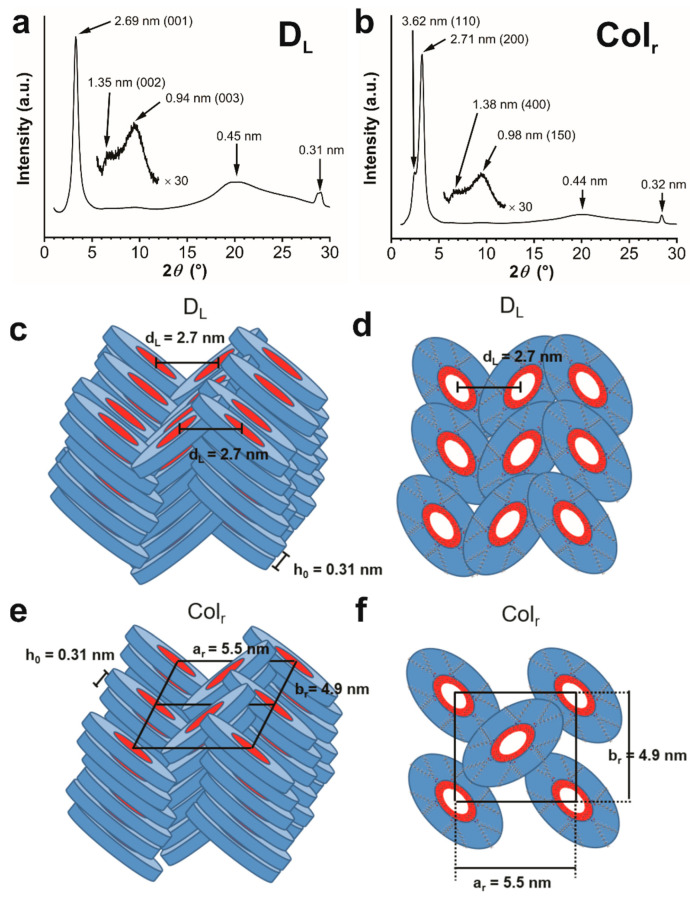
PXRD patterns of representative (**a**) lamello-columnar (D_L_) and (**b**) rectangular columnar (Col_r_) phases in aerogels of **ISA24** randomly observed from freeze-dried cyclohexane organogels (2 wt%). Schematic representation of the proposed columnar phases of **ISA24.** (**c**) Side and (**d**) top views of the lamello-columnar phase (D_L_). (**e**) Side and (**f**) top view of the rectangular columnar phase (Col_r_). *d*_L_ = repeating interlayer distance. *h*_0_ = distance between discoids. *a*_r_, *b*_r_ = Col_r_ phase unit cell parameters.

Powder X-ray diffraction (PXRD) was used to gain further insight into the molecular packing of **ISA24** within the dried gel phases. All PXRD patterns were characteristic of ordered, discotic columnar materials (Figure 8 and Figure S33) [95,96], which is consistent with the 1D aggregates observed in microscopy images (Figure 6 and Figure 7), i.e., stacks of cyclic, H-bonded hexamers (Figure 1). One or more strong Bragg reflections appear at low angles (2*θ* < 4°), while multiple weak, higher-order reflections occur at intermediate angles (4° < 2*θ* < 16°), as well as a broad halo and sharp, well-defined peaks at wide angles (2*θ* > 16°) [95,96]. The intense reflections at low angles/large *d*-spacings correspond to intercolumnar distances, while the broad halo in the wide-angle region is characteristic of the liquid-like ordering of alkyl chains (*h*_ch_) [95,96]. The sharp, well-defined peak at ~0.31 nm is typical of long-range core–core π–π stacking (*h*_0_) within columns of flat molecular cores (i.e., cyclic hexamer aggregates here, Figure 1) [95,96]. The inter-disk distance of 0.31 nm here is significantly small for an assumed π–π stacking, which usually falls within the range 0.35–0.36 nm and is consistent with the formation of elongated fiber-like aggregates. H-bonding between the amide functional linker groups is likely responsible for the observed decrease in the inter-disk distance, as previously shown for other disk-shaped compounds with peripheral alkyl amide groups [97].

All of the PXRD patterns for **ISA24** were indexed using *LCDiXRay* [95] and assigned to either lamello-columnar (D_L_) or rectangular columnar (Col_r_) phases. Figure 8a shows an example of a PXRD pattern of the D_L_ phase of **ISA24**, which exhibits five reflections at approximately 2.7 (*001*), 1.3 (*002*), 0.9 (*003*), 0.45 (*h*_ch_), and 0.31 nm (*h*_0_). The ratio of the reciprocal *d*-spacings of the first three reflections is 1:2:3, which indicates a pronounced layered structure with a repetitive distance, *d*_L_, of ~2.7 nm. This value of *d*_L_ is small relative to the calculated diameter of cyclic hexamer disks from **ISA24** (Table S5 and Figure S33). Therefore, the disks are probably tilted approximately 60° to accommodate enough space for the alkyl tail-groups while maintaining the relatively short *d*_L_ of ~2.7 nm (Figure 8c,d). Tilting of the cyclic hexamer disks within the columns relative to the column long axis may also account for the slight difference measured between the AFM cross-section height (~3.6 nm) and the HR-TEM width (~4.5 nm). The D_L_ systems were also observed for the dried gels of **ISA24** from other solvents such as toluene and CHCl_3_ (Figure S33, Table S5).

Figure 8b shows an example of a PXRD pattern assigned to the Col_r_ phase of **ISA24** in an ex situ, freeze-dried aerogel from cyclohexane (2 wt%). Six peaks are present at 3.62 (*110*), 2.71 (*200*), 1.38 (*400*), 0.98 (*150*), 0.44 (*h*_ch_), and 0.32 (*h*_0_) nm, from which the unit cell parameters of *a*_r_ = 5.42 nm and *b*_r_ = 4.86 nm were calculated. A schematic representation of the Col_r_ phase from **ISA24** with the lattice parameters is shown in Figure 8e,f. Col_r_ phases of **ISA24** were also observed from a xerogel and aerogel from benzene as well as xerogels from cyclohexane (Figure S33 and Table S6). Changes in the intensities and *d*-spacings of the peaks were observed, which indicate different lattice dimensions due to shrinkage [98]. While the method of drying clearly effects the morphology of the gel network structures formed, it does not appear to effect the supramolecular organization of **ISA24**. Both D_L_ or Col_r_ phases from cyclohexane gels were produced randomly in the xerogels or aerogels (Figure 8 and Figure S33, Tables S5 and S6), which leads us to believe that other conditions are responsible (i.e., solvent type, gelator concentration, gelation temperature, equilibration time, cooling rate, solvent evaporation rate, freezing rate and time, etc.).

### 2.10. Phase-Selective Gelation

Phase-selective organogelators (PSOGs) of organic solvents from biphasic mixtures with water by LMWGs have attracted significant interest over the last decade for the clean-up of marine oil spills [22,23,55,56,57]. Such technologies have the potential to prevent the flow of organic liquids over water bodies as well as facilitate removal from otherwise liquid–liquid mixtures. The technical requirements for oil spill clean-up include high gelator solubility in a carrier liquid, efficient gelator ability, rapid gelation at room temperature, low toxicity, and low cost [22,23].

The excellent gelator ability of **ISA24** with low polarity organic solvents motivated us to explore its potential as a PSOG for solvent:water mixtures. In principle, the phase-selective gelation of oil in the presence of water may be challenging due to water’s strong ability to compete for H-bonding sites in the gelator molecules. The PSOG ability of **ISA24** towards kerosene and crude oil was tested for biphasic mixtures with water using the “vial inversion” test after heating the samples at ~100 °C, followed by cooling and equilibration at 23 °C for at least 30 min. The kerosene gel phase (~0.5 wt%) was able to hold its own weight in addition to the weight of the top aqueous phase (Figure S34), whereas the crude oil gel phase (~1 wt%) was unstable and fell during vial inversion tests. The presence of water apparently only affected the integrity of the crude oil gel.

Since heating would be impractical for industrial-scale spills, three other methods to trigger the gelation of kerosene from kerosene:water mixtures by **ISA24** were also explored. First, using the co-solvent method, a 0.2 M solution of **ISA24** in methanol (0.2 mL) was added to a 1:1 kerosene:water mixture (2 mL). After 24 h, the kerosene phase was selectively gelled by **ISA24** (2 wt%), while the liquid water phase remained separate. The second method involved the addition of bulk **ISA24** powder (9.6 mg) to a 1:1 kerosene:water mixture (2 mL), followed by vortex mixing for 5 min. Although the solid did not completely dissolve after vortex mixing, the kerosene phase did gel selectively after 24 h (1 wt%). Finally, bulk **ISA24** powder (10.4 mg) was also added to a 1:1 kerosene:water mixture (2 mL), followed by ultrasonic bath mixing for 5 min. Again, although **ISA24** was not completely solubilized, the top kerosene layer formed a gel within 5 min (1 wt%). All three kerosene gels formed using a co-solvent, vortex mixing, or ultrasonic bath mixing resulted in organogels that were strong enough to hold their own weight as well as the liquid aqueous phase. The PSOG behavior for **ISA24** suggests the potential use for marine oil spill clean-up.

### 2.11. Dye Removal from Water

**Figure 9:**
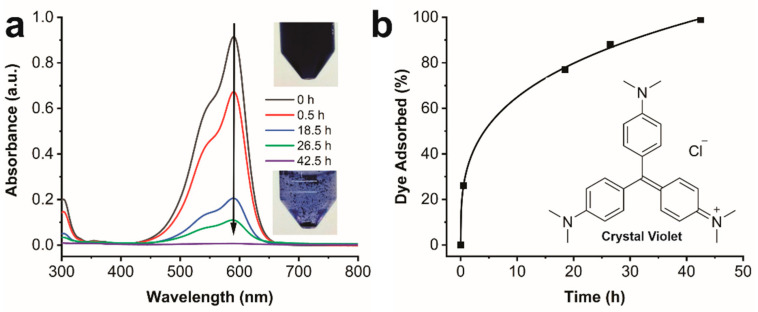
Crystal Violet (CV) dye adsorption by the **ISA24** aerogel. (**a**) UV-Vis spectra of CV recorded after various times. (**b**) The amount of CV dye adsorbed by the **ISA24** aerogel over time.

Recently, gel-based materials from LMWGs have demonstrated promise as solid phase adsorbents for the removal of dyes from contaminated water [45,99]. Organogel networks have low solubility in water at low temperatures, high porosity, low density, and high internal surface area allowing easy diffusion of molecular species of interest within their structures. A variety of LMWGs have been reported to adsorb dyes from aqueous solutions at rates ranging from minutes to several days [23,58,59,60]. Considering the 3D network structure, **ISA24** was expected to be an excellent adsorbent for the removal of dyes from water. Therefore, the dye removal ability of the 3D gel network from **ISA24** was evaluated for the toxic cationic dye Crystal Violet (CV, Figure 9).

Dye adsorption was examined by adding aerogel powder of **ISA24** to an aqueous solution of Crystal Violet (CV). Over time, the color of the freeze-dried gel visually changed from white to violet, and the solution went from violet to colorless and transparent as the dye was efficiently adsorbed (Figure 9). Aliquots were removed over time and analyzed by UV-Vis spectroscopy (Figure 9), which showed that the UV-Vis band of CV between 450–650 nm decreased over time and disappeared completely after 43 h (Figure 9a). After 43 h, nearly all of the dye was adsorbed, and the absorption capacity was calculated to be ~90 mg/g. Therefore, these results indicate that the gel from **ISA24** is efficient for the adsorption of cationic dyes, such as CV from water. Similar experiments were also carried out using the “wet” organogels from **ISA24** with cyclohexane; however, the rate of adsorption was significantly slower due to the lower surface area in contact with the aqueous phase. The transfer of CV from the aqueous phase to the cyclohexane oil phase did not occur in the absence of **ISA24**. The effectiveness of CV dye absorption from water demonstrated that 5-alkylamido ISA derivatives such as **ISA24** could play an important role in the removal of contaminants from polluted water or industrial water treatments.

## 3. Conclusions

In summary, this work focused on how to design new LMWGs based on the ISA supramolecular synthon via a highly efficient synthesis method. These new alkylamido ISA gelators were highly efficient organogelators, especially for low polarity organic solvents, as evidenced by their supergelator ability for many solvents. We demonstrated that the gelation region(s) in Hansen space and stability of the gel over time can be tuned with changes to the tail-group chemical structure. Optimized gelator performance was achieved for the branched **ISA24** derivative, gelling a wide variety of solvents at low concentrations, which is noteworthy. Unlike for previously reported BZI gelators [67], branched tail-groups were not required for gelation. Microscopy, spectroscopy, and PXRD data were all consistent with the formation of nanofibers from stacks of cyclic, H-bonded hexamers and a self-assembly process driven by H-bonding, π–π stacking, and van der Waals interactions. For low polarity solvents, the nature of the solvent plays a significant role in bundling of the nanofibers and the resulting SAFIN morphology. Finally, the selective gelation of an oil phase from oil: water mixtures and dye adsorption experiments demonstrated the potential of these compounds for water purification applications such as oil spill clean-up and as solid phase adsorbents for removing soluble contaminants from contaminated water, respectively. This work is anticipated to inspire the further design of alkylated ISA supramolecular chemistry as well as enrich the rapid development of alkylated ISA multifunctional materials.

## 4. Materials and Methods

### 4.1. General Information

All chemicals were commercially available and used without further purification. ISOCARB 12 (2-butylhexanoic acid), ISOCARB 16 (2-hexyldecanoic acid), and ISOCARB 24 (2-decyltetradecanoic acid) were acquired from Sasol America (Houston, TX, USA). Syncrude sweet crude oil was acquired from Syncrude (Edmonton, AB, Canada). Gasoline (regular unleaded) was acquired from a local Safeway gas station. Motor oil (Tech 2000, SAE 10W30) was acquired from the local market. *N,N*-dimethylformamide (anhydrous, >99.8%) was acquired from Acros. 3-bromohexanoyl chloride and hexadecane were acquired from TCI America (Portland, OR, USA). CDCl_3_ (D-99.8%) and DMSO-*d*_6_ (D-99%) were used as received from Cambridge Isotope Laboratories (Tewksbury, MA, USA). Potassium hydroxide (85%), sodium chloride, dimethyl sulfoxide, potassium iodide, ethylene glycol, methanol, acetone, ethyl acetate, diethyl ether, chloroform, benzene, hexanes, silicon oil, and olive oil were acquired from Fisher Scientific. All other chemicals were acquired from the Sigma-Aldrich chemical company (Mississauga, ON, Canada). Deionized (DI) water was purified using a Milli-Q ultrapure unit.

### 4.2. Synthesis and Characterization of Gelators ISA4, ISA16L, ISA12, ISA16, and ISA24

The details are presented in the Supporting Information. All reactions were performed under a nitrogen atmosphere using standard Schlenk techniques unless otherwise stated.

### 4.3. Nuclear Magnetic Resonance (NMR) Spectroscopy

^1^H and ^13^C NMR spectra were recorded with a Varian AMX600 spectrometer. Chemical shifts (*δ*) are in ppm. Multiplicities are denoted as follows: s = singlet, m = multiplet, br = broad. 

### 4.4. Mass Spectrometry (MS)

High-resolution electrospray ionization (HR-ESI) mass spectra were acquired using an Agilent Technologies 6220 orthogonal acceleration time-of-flight (oaTOF) instrument in negative ion mode.

### 4.5. Gelation Test

The gelation behaviors of the 5-alkylated ISA compounds were examined using the “vial inversion test” [24]. The procedure involved placing the 5-alkylated ISA compound (1–100 mg) and a liquid (1 mL) into a glass vial (4 mL, outer diameter = 15 mm, height = 45 mm) with a threaded top, which was sealed with Teflon tape and a screw-cap lid. The mixture was then immersed in an ultrasonic bath (Bransonic 3510R-MT, 117 V, 50–60 Hz) until a fine suspension was obtained, which required times ranging from 5 s to 30 min. The suspension was then heated to the liquid boiling temperature until the solid fully dissolved and a transparent, clear solution was achieved. The mixtures were then slowly cooled to 23 °C. After 30 min at 23 °C, the vial was inverted in order to test whether or not the resulting mixture visibly flowed or dropped to the bottom of the inverted the vial due to its weight influenced by gravity (i.e., the vial inversion test). The gels were designated as clear (CG), turbid (TG), and opaque (OG). Samples that did not form clear solutions with heating were characterized as insoluble (I), while samples that formed clear solutions with heating but precipitated with cooling were labeled precipitates (P). Mixtures that remained as a clear solution were categorized as solution (S). The critical gel concentrations (*CGC*s) were determined by carrying out a series of vial inversion tests at different gelator concentrations, which typically involved gelator increments of 1–2 mg/mL (or 0.1–0.2 wt%). The *CGC* was the lowest gelator concentration at which the sample did not flow or fall at 23 °C.

### 4.6. Hansen Solubility Parameters

HSPs were initially developed to explain the solubility of polymers in different solvents [75]. This concept was later applied by Raynal and Boutellier to reason why molecular gelators gel some solvents and not others [71,74]. Solvents were defined by three types of HSP interactions, which include dispersive (δ_d_), H-bonding (δ_h_), and polar interactions (δ_p_), and were obtained through the HSPiP software [75] and the literature. The results of the gelation tests in various solvents and their HSPs [73,75] of **ISA4**, **ISA16L**, **ISA16**, and **ISA24** are summarized in Table S2. Figure S16 shows the results of the gelation tests plotted in 3D Hansen space. Green, blue, and red data points indicate gels, solutions, and insoluble/precipitates, respectively. The HSP and radius for each gelation sphere were determined by the genetic algorithm, single or double sphere-fit method using HSPiP software [63,68]. Since ethylene glycol was the only data point in second sphere region (high δ_p_ and δ_h_ values), the second gelation sphere calculated by the HSPiP was ignored and was taken to be a single point with no radius.

### 4.7. Gel-to-Sol Transition Temperature Measurements

Gel-to-sol transition temperatures (*T*_gel_) were determined using the “falling ball” method [100]. A 2 mm diameter stainless steel ball (33.2 ± 0.1 mg) was placed on the top of the gel in a sealed screw-cap vial. The vial was immersed in a silicon oil bath and the temperature was slowly increased (1–2 °C/min). *T*_gel_ was accepted to be the temperature at which the ball touched the bottom of the vial.

### 4.8. Rheological Measurements

Rheological data were acquired using a Discovery hybrid rheometer HR-3 (TA instruments, New Castle, DE, USA) equipped with a parallel plate geometry with a 40 mm diameter cross-hatched plate (used gap = 1 mm) and a Peltier system for temperature control to measure the elastic modulus (*G*′, storage modulus) and the viscous modulus (*G*″, loss modulus). The gel samples were prepared ex situ in vials and transferred to the lower Peltier plate using a spatula. All measurements were carried out in triplicate at 25 °C. Strain sweep measurements were performed between 0.01–100% at a frequency of 1 Hz in order to measure the mechanical strengths of the gels and define the linear visco-elastic region (LVR). Frequency sweep measurements were carried out over the frequency range of 1–100 rad/s at amplitude strain values of 0.1, 0.5, 1, 2, and 2 wt% for the gels with xylenes, cyclohexane, crude oil, toluene, and decalin, respectively.

### 4.9. In Situ Aerogel Preparation (for cryo-SEM)

In situ refers to samples that were prepared at small scale (i.e., <0.1 mL) directly on microscopy sample substrates. A piece of organogel was placed between two copper rivets and rapidly frozen by plunging into liquid N_2_ for five min. The frozen organogel was then fractured to reveal the frozen surface, and the frozen solvent was sublimed at −100 °C for 30 min. The resulting fractured aerogel surface was then sputter-coated with a Pt film for 120 s (thickness ~5 nm) using a cryo-coater (Leica ACE600).

### 4.10. Ex Situ Aerogel Preparation

Ex situ refers to aerogel samples that were prepared at large scale (i.e., 1–20 mL) and later transferred onto microscopy sample substrates. Organogels (1–20 mL, 2 wt%) in glass vials were rapidly frozen by plunging into liquid N_2_. After cooling for 30 min, the frozen organogels were then freeze-dried at −50 °C and 0.01 mbar (Labconco FreeZone Freeze Dryer). The frozen organogels were dried for 72 h to give solid aerogel monoliths that had approximately the same volume of the precursor wet gel but were not crack free.

### 4.11. Thick Xerogel Film Preparation

Thick xerogel films were deposited using the most common method involving drying of the gel [79]. A small piece of gel was placed onto the substrate and air-dried at 23 °C for 16 h or longer.

### 4.12. Thin Xerogel Film and Self-Assembled Nanostructure Synthesis

Thin xerogel films and self-assembled nanostructures were prepared from respective solutions of a 5-alkylamido ISA dissolved in solvents at concentrations precluding gel formation. A droplet of the solution (5–10 μm) was deposited onto the substrate and the excess liquid was removed by blotting with filter paper (Whatman #1) to generate a thin film that was air-dried at 23 °C for 0.5–24 h.

### 4.13. Polarized Optical Microscopy (POM)

Polarized optical microscope (POM) images were acquired using a Zeiss Axio Scope A1 in different contrast and polarization modes. All samples were examined on glass microscope slides.

### 4.14. Scanning Electron Microscopy (SEM)

All SEM samples were deposited onto ultra-thin carbon-coated copper TEM grid substrates (400 mesh, Electron Microscopy Sciences (EMS), Hatfield, PA, USA). Thick xerogel samples and ex situ aerogels were coated with gold/platinum (5 nm) of using a Gatan 682 PECS Ion Beam Sputtering and Etching System prior to analysis. Images were acquired using a field emission scanning electron microscope (Hitachi S-4800 or S-5500 FE-SEM) operating at 5–30 kV. Ex situ aerogel powders were applied to TEM grids coated with carbon paint. 

### 4.15. High-Resolution Transmission Electron Microscopy (HR-TEM)

Nanofibrils of **ISA24** deposited from a solution of **ISA24** in cyclohexane (0.2 mg/mL) were negatively stained with a droplet (10 μL) aqueous uranyl acetate (2 wt%) on the TEM sample grid. After 120 s, the excess uranyl acetate solution was blotted away with filter paper (Whatman #1) and air-dried on a hot plate (40 °C, 1 h) to produce a solid thin film. HR-TEM images were acquired using a high-resolution transmission electron microscope (JEOL 2200 FS TEM—200 kV Schottky field emission instrument equipped with an in-column omega filter). Bright field images were obtained using energy filtered zero loss beams (slit width of 10 ev).

### 4.16. Cryo-Scanning Electron Microscopy (cryo-SEM)

In situ, freeze-fractured aerogel samples were imaged using a Zeiss NVision40 SEM at −140 °C, using an accelerating voltage of 3 keV and an in-lens energy and angle selective backscattered electron (EsB) detector.

### 4.17. Atomic Force Microscopy (AFM)

All samples for AFM examination were deposited and dried onto freshly cleaved mica substrates (1 cm^2^, Ted Pella, Redding, CA, USA). AFM images were acquired using a Digital Instruments/Veeco Instruments MultiMode Nanoscope IV atomic force microscope equipped with an E scanner. Silicon cantilevers (MikroMasch, Portland, OR, USA) with low spring constants (4.5 N/m) were used in tapping mode at low scan rates (0.5–1 Hz) and an amplitude set point of 1 V for optimum height profiles. All images were flattened to remove the background curvature of substrate surfaces. The structures observed in the AFM images are assumed to be related to those in the native wet gel state, which may or may not be true [79,86].

### 4.18. Computer Modeling

The geometries of cyclic hexamers were optimized in the gas phase using the Polak–Ribiere conjugate gradient algorithm of the Hyperchem 7.51 program (Hyperchem, Hypercube Inc., Gainsville, FL, USA) through semi-empirical calculations, using the AM1 method with an RMS gradient of 0.01 kcal/Å mol [67].

### 4.19. Attenuated Reflectance Fourier Transform Infrared Spectroscopy (ATR-FTIR) Spectroscopy

FTIR spectra were recorded at 23 °C using a Digilab FTS 7000 Series spectrometer equipped with am Attenuated Total Reflectance (ATR) accessory (Harrick MVP-pro).

### 4.20. Powder X-ray Diffraction (PXRD)

PXRD data were acquired between 1° ≤ 2 Theta ≤ 30° (step size = 0.01°) with a Bruker model D8/Discover X-ray diffractometer using CuKa radiation (λ = 1.542 Å) at 50 kV and 10 mA. The resulting data were analyzed using EVA^tm^ software. All PXRD patterns were indexed using *LCDiXRay* [95] software.

### 4.21. Crystal Violet (CV) Adsorption

CV adsorption was performed by adding **ISA24** aerogel powder to an aqueous solution of CV. The adsorption of CV over time was monitored using a Perkin-Elmer UV-Vis spectrophotometer. Typically, the aerogel solid (4 mg) was added to an aqueous solution of CV (0.1 mM, 10 mL). After each time interval, an aliquot (0.2 mL) was removed and diluted to 2 mL for UV-Vis examination. The amount of dye absorbed (DA, %) was calculated using the equation:

DA = (1 − (*c_i_* − *c_t_*)/*c_i_*) × 100

where *c_i_* and *c_t_* are the initial and concentrations after time *t* of CV, respectively. The maximum adsorption capacity, *Q* (in mg of CV/g of aerogel), after the longest *t* was calculated using the equation:

*Q* = (*c_i_* − *c_t_*) *V/m*

where *V* is the volume (in L) and *m* is the mass (in g) of the dried gel powder.

## Data Availability

Not applicable.

## Supplementary Materials

The following supporting information can be downloaded at: https://www.mdpi.com/article/10.3390/gels8050285/s1, synthesis procedures for **ISA4**, **ISA16L**, **ISA12**, **ISA16**, and **ISA24**; Scheme S1. Synthesis of 5-alkanamido ISA derivatives; Figures S1–S3: ^1^H NMR, ^13^C NMR and HRMS spectra of **ISA4**; Figures S4–S6: ^1^H NMR, ^13^C NMR and HRMS spectra of **ISA16L**; Figures S7–S9: ^1^H NMR, ^13^C NMR and HRMS spectra of **ISA12**; Figures S10–S12: ^1^H NMR, ^13^C and HRMS spectra of **ISA16**; Figures S13–S15: ^1^H NMR, ^13^C NMR and HRMS spectra of **ISA24**. Table S1: Appearances of ISA gelators in different liquids and CGC values (wt%) of the gels; Table S2: Hansen solubility parameters (MPa^1/2^) for and gelation tests with various solvents for 5-alkylamido ISA derivatives (1 wt%); Figure S16: Solubility data for organogelators **ISA12**, **ISA16**, **ISA16L**, and **ISA24** represented in Hansen space; Figure S17: SEM images of nanofibrils deposited from 5-alkylated ISA derivatives; Figure S18: SEM images of self-assembled nanostructures of ISA4 deposited from solutions; Figure S19: SEM images of xerogels, SAFINs, self-assembled nanofibers of 5-alkylamido ISAs deposited from toluene solutions (0.1 mg/mL); Figure S20: SEM images of SAFINs of **ISA12**, **ISA16**, and **ISA16L** deposited from xylenes: THF (19:1) solutions; Figure S21: SEM images of self-assembled nanostructures of **ISA24** deposited from cyclohexane; Figure S22: SEM images of self-assembled nanostructures of **ISA24** deposited from toluene; Figure S23: SEM images of self-assembled nanostructures of **ISA24** deposited from CHCl3; Figure S24: SEM images of self-assembled nanostructures of **ISA24** deposited from THF; Figure S25: HR-TEM images of nanofibrils from **ISA24** formed in cyclohexane (0.02 wt%); Figure S26: AFM height images of nanofibrils formed by **ISA24** in cyclohexane (0.02 wt%) on freshly cleaved mica; Table S4: Measured nanofiber widths and cyclic hexamer diameters from computer models (Figure S20) for alkylamido ISA derivatives; Figure S27: Ball-and-stick computer models of cyclic hexamers from 5-alkylamido ISAs and their approximate maximum diameters; Figure S28: POM images of gels of **ISA24** with different solvents; Figure S29: Cryo-SEM images of **ISA24** from cyclohexane (2 wt%); Figure S30: FE-SEM images of xerogels of **ISA24** from different solvents; Figure S31: ^1^H NMR spectra of **ISA24**; Figure S32: Partial ATR-FTIR spectra of **ISA24**; Figure S33: PXRD patterns of **ISA24**; Table S5: Powder X-ray diffraction data of lamello-columno phases of **ISA24** from dried gel samples; Table S6: Powder X-ray diffraction data of rectangular columnar (Col_r_) phases of **ISA24** from dried gel samples; Figure S34: Digital photos of the phase selective gelation by **ISA24**.
