# Supplementary material for: Self-Assembly of Alkylamido Isophthalic Acids toward the Design of a Supergelator: Phase-Selective Gelation and Dye Adsorption
Source: Gels. 2022 May 5;8(5):285. doi: 10.3390/gels8050285 (PMC9140382; doi:10.3390/gels8050285)
Supplement: Supplementary file 1 [file gels-08-00285-s001.zip › gels-1665237-supplementary.pdf]

Supporting Information for

Self-Assembly of Alkylamido Isophthalic Acids Toward the Design of a Supergelator: Phase Selective Gelation and Dye Adsorption

Darren A. Makeiff[a]* Jae-Young Cho,[a] Bradley Smith,[a] Rina Carlini[b] and Nicolas Godbert[c]

[a]Nanotechnology Research Center, National Research Council of Canada, 11421 Saskatchewan Drive, Edmonton, Alberta, Canada, T6G 2M9, E-mail: Darren.Makeiff@nrc.ca

[b]Optimal Innovation Group Inc, 175 Longwood Rd. S, suite 112 A, Hamilton, Ontario, Canada, L8P 0A1

[c]Dipartimento di Chimica e Tecnologie Chimiche, Università della Calabria, 87036 Arcavacata di Rende (CS) (Italy)

Contents

Synthesis Procedures.......... 4

Scheme S1. Synthesis of 5-alkanamido ISA derivatives.......... 5

Synthesis of ISA4′.......... 5

Synthesis of ISA4.......... 6

Figure S1. 600 MHz ^{1}H NMR spectrum of **ISA4** in DMSO-d_6.......... 7

Figure S2. 150 MHz ^{13}C NMR spectrum of **ISA4** in DMSO-d_6.......... 7

Figure S3. High Resolution Mass Spectrum of **ISA4**.......... 8

Synthesis of ISA16L′.......... 8

Synthesis of ISA16L.......... 9

Figure S4. 600 MHz ^{1}H NMR spectrum of **ISA16L** in DMSO-d_6.......... 10

Figure S5. 150 MHz ^{13}C NMR spectrum of **ISA16L** in DMSO-d_6.......... 11

Figure S6. High Resolution Mass Spectrum of **ISA16L**.......... 11

Synthesis of ISA12′.......... 11

Synthesis of ISA12.......... 12

Figure S7. 600 MHz ^{1}H NMR spectrum of **ISA12** in DMSO-d_6.......... 13

Figure S8. 150 MHz ^{13}C NMR spectrum of **ISA12** in DMSO-d_6.......... 14

Figure S9. High Resolution Mass Spectrum of **ISA12**.......... 14

Synthesis of ISA16.......... 15

Figure S10. 600 MHz ^{1}H NMR spectrum of **ISA16** in DMSO-d_6.......... 17

Figure S11. 150 MHz ^{13}C NMR spectrum of **ISA16** in DMSO-d_6.......... 17

Figure S12. High Resolution Mass Spectrum of **ISA16**.......... 18

Synthesis of ISA24'.......... 18

Figure S13. 600 MHz ^{1}H NMR spectrum of **ISA24** in DMSO-d_6.......... 20

Figure S14. 150 MHz ^{13}C NMR spectrum of **ISA24** in DMSO-d_6. 21

Figure S15. High Resolution Mass Spectrum of **ISA24**. 21

Gelation Behavior 23

Table S1. Appearances of ISA gelators in different liquids and CGC values (wt %) of the gels 23

Hansen Solubility Parameters 25

Table S2. Hansen solubility parameters ($MPa^{1/2}$) for and gelation tests with various solvents for 5-alkylamido ISA derivatives (1 wt %) 25

Figure S16. Solubility data for organogelators **ISA12**, **ISA16**, **ISA16L**, and **ISA24** represented in Hansen space. 27

Gel Characterization 28

Figure S17. SEM images of nanofibrils deposited from 5-alkylated ISA derivatives. 28

Figure S18. SEM images of self-assembled nanostructures of **ISA4** deposited from solutions. 29

Figure S19. SEM images of xerogels, SAFINs, self-assembled nanofibers of 5-alkylamido ISAs deposited from toluene solutions (0.1 mg/mL). 29

Figure S20. SEM images of SAFINs of **ISA12**, **ISA16**, and **ISA16L** deposited from xylenes : THF (19:1) solutions. 30

Figure S21. SEM images of self-assembled nanostructures of **ISA24** deposited from cyclohexane. 30

Figure S22. SEM images of self-assembled nanostructures of **ISA24** deposited from toluene. 31

Figure S23. SEM images of self-assembled nanostructures of **ISA24** deposited from $CHCl_3$. 31

Figure S24. SEM images of self-assembled nanostructures of **ISA24** deposited from THF 32

Figure S25. HR-TEM images of nanofibrils from **ISA24** formed in cyclohexane (0.02 wt %). 32

Figure S26. AFM height images of nanofibrils formed by **ISA24** in cyclohexane (0.02 wt %) on freshly cleaved mica. 33

Table S4. Measured nanofiber widths and cyclic hexamer diameters from computer models (Figure S20) for alkylamido ISA derivatives. 33

Figure S27. Ball-and-stick computer models of cyclic hexamers from 5-alkylamido ISAs and their approximate maximum diameters. 34
Figure S28. POM images of gels of **ISA24** with different solvents. 35
Figure S29. Cryo-SEM images of **ISA24** from cyclohexane (2 wt %)........ 35
Figure S30. FE-SEM images of xerogels of **ISA24** from different solvents. 36
Figure S31. ^{1}H NMR spectra of **ISA24**........ 37
Figure S32. Partial ATR-FTIR spectra of **ISA24**. 38
Figure S33. PXRD patterns of **ISA24**. 39
Table S5. Powder X-ray diffraction data of lamello-columno phases of **ISA24** from dried gel samples. 40
Table S6. Powder X-ray diffraction data of rectangular columnar (Col_r) phases of **ISA24** from dried gel samples........ 41
Figure S34. Digital photos of the phase selective gelation by **ISA24**........ 42

Synthesis Procedures

The 5-alkylamide isophthalic acid (ISA) compounds **ISA4**, **ISA12**, **ISA16**, **ISA16L**, and **ISA24** were synthesized in two to three steps according to Scheme S1. First, dimethyl 5-aminoisophthalate (**DMAISA**) was coupled with the respective acid chlorides **1**, **2**, **6**, **7**, and **8** to give the corresponding dimethyl ester compounds **ISA4'**, **ISA12'**, **ISA16'**, **ISA16L'**, and **ISA24'**. Note that the acid chlorides **1** and **2** were commercially available, while **6-8** were prepared from the corresponding commercially available acids **3-5** were synthesized using oxalyl chloride in THF with a catalytic amount of DMF. The free diacid compounds **ISA4**, **ISA12**, **ISA16**, **ISA16L**, and **ISA24** were obtained in excellent overall yield after saponification of the respective diesters with KOH in methanol-water, followed by acidification with HCl.

Scheme S1. Synthesis of 5-alkanamido ISA derivatives

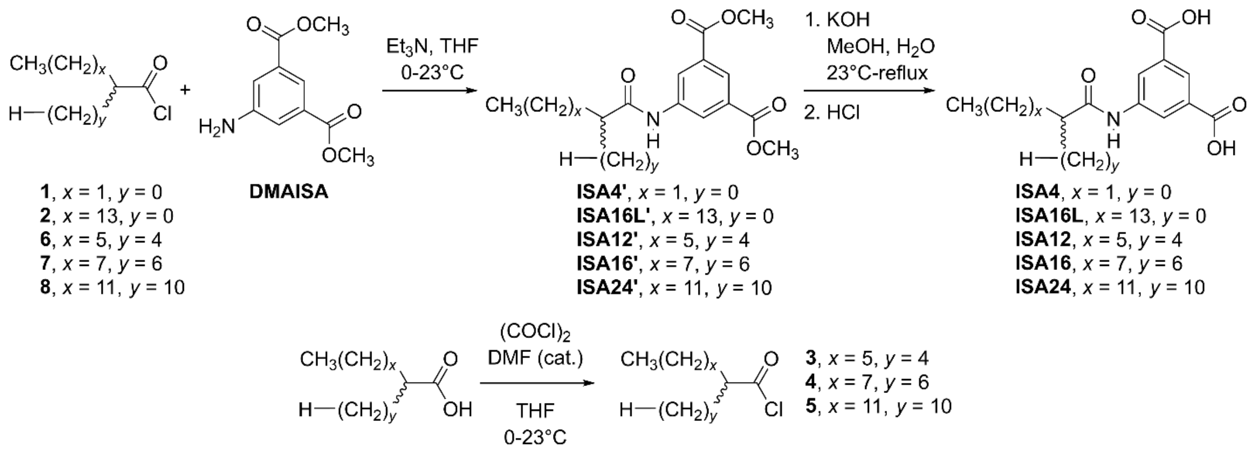

Synthesis of ISA4′

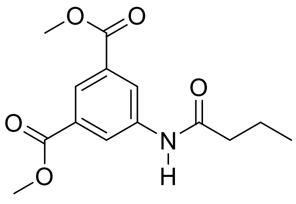

Butyryl chloride **1** (0.4 mL, 3.82 mmol) was added slowly, dropwise to 5-amino-1,3-dimethyl isophthalate (0.78 g, 3.67 mmol), triethylamine (1 mL, 7.17 mmol), and dry THF (20 mL) at 0°C. The reaction mixture was then slowly allowed to warm to 23°C and stirred overnight. Deionized water (20 mL) was then added, and the mixture was extracted with diethyl ether (50 mL). The ether layer was separated, washed successively with 10 mL portions of saturated sodium bicarbonate, deionized water, 5% citric acid, deionized water, and brine, and then dried over sodium sulfate and filtered. Rotary evaporation of

the excess ether and drying *in vacuo* afforded dimethyl 5-butyramidoisophthalate **ISA4′** as a white solid (1.02 g, 100%). **^{1}H NMR** (600 MHz, $CDCl_3$, ppm) δ 8.41 (m, 2H), 8.37 (m, 1H), 8.05 (br s, 1H), 3.90 (s, 6H), 2.37 (t, J = 7.5 Hz, 2H), 1.76 (m, 2H), 0.98 (t, J = 7.5 Hz, 3H). **^{13}C NMR** (150 MHz, $CDCl_3$, ppm) δ 172.1, 166.2, 138.9, 131.4, 126.2, 125.1, 52.6, 39.6, 19.1, 13.9.

Synthesis of ISA4

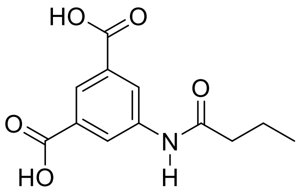

ISA4′ (1.02 g, 3.65 mmol), KOH (2.06 g, 35.60- mmol), and methanol (20 mL) were heated to reflux. After 1 h, the reaction mixture was cooled to 23°C to give a thick slurry that was acidified with 5 M HCl. The slurry was then filtered, washed with deionized water and dried in vacuo to afford **ISA4** as a white solid (0.77 g, 84%). **^{1}H NMR** (600 MHz, DMSO-*d_6*, ppm) δ 10.23 (s, 1H), 8.44 (s, 2H), 8.14 (s, 1H), 2.31 (t, J = 8.4 Hz, 2H), 1.60 (m, 2H), 0.89 (t, J = 7.5 Hz, 3H). ^{13}C NMR (150 MHz, DMSO-*d_6*, ppm) δ 171.8, 166.6, 140.0, 131.7, 124.4, 123.6, 38.4, 18.5, 13.6. **HRMS** (EI, M^-) cald for $C_{12}H_{12}NO_5$ 250.0721 $(M-H)^-$, 250.0718 $(M-H)^-$ found.

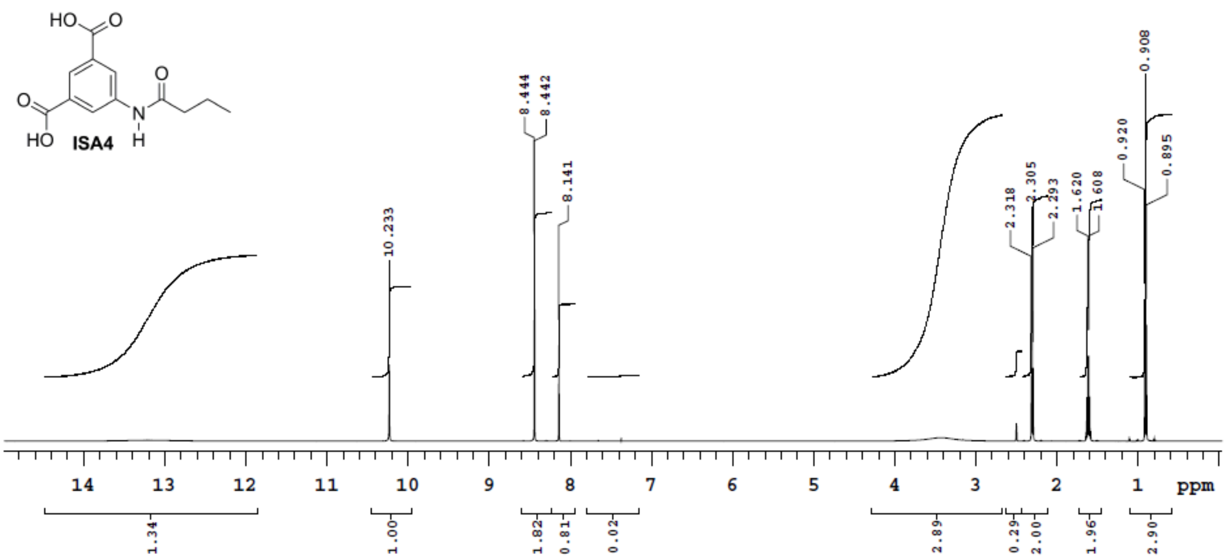

Figure S1. 600 MHz ^{1}H NMR spectrum of **ISA4** in DMSO-d_6.

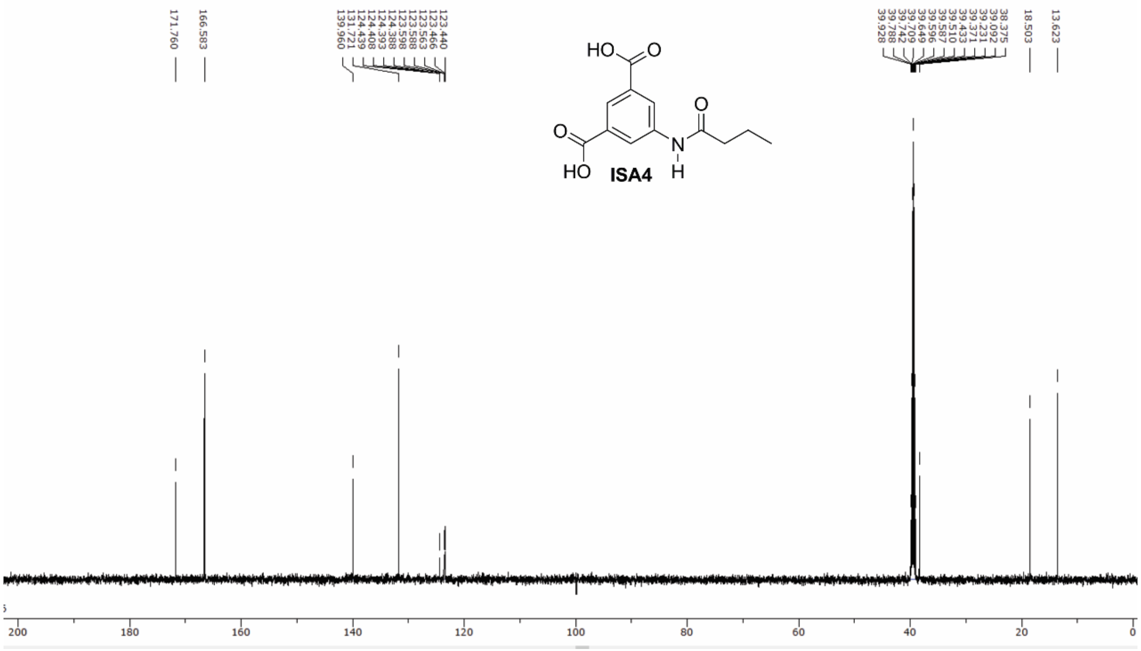

Figure S2. 150 MHz ^{13}C NMR spectrum of **ISA4** in DMSO-d_6.

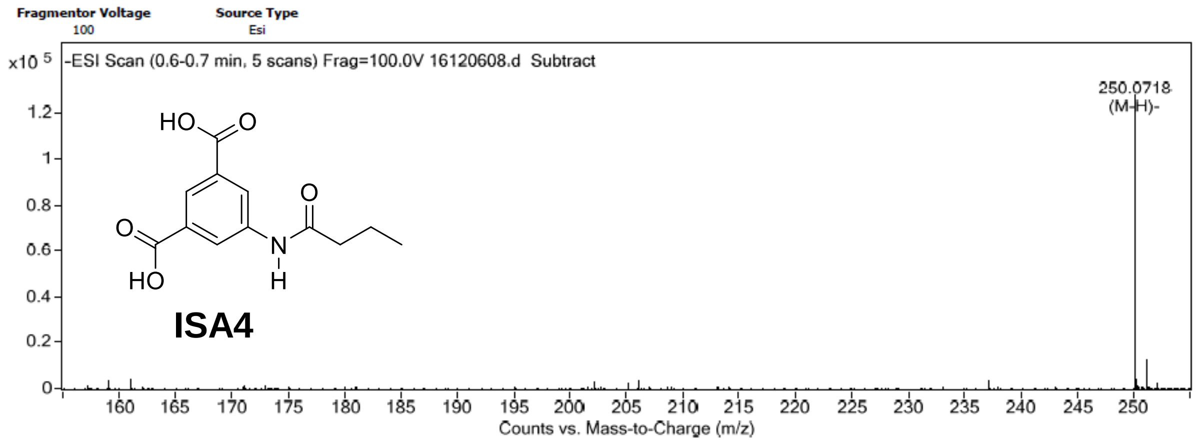

Formula Calculator Results

Formula	Ion Species	Mass	Calc. Mass	m/z	Calc. m/z	Diff (mDa)	Diff(ppm)	DBE	Ion	Score
C12 H13 N O5	C12 H12 N O5	251.0791	251.0794	250.0718	250.0721	0.25	0.99	7	(M-H)-	83.95

Figure S3. High Resolution Mass Spectrum of **ISA4**.

Synthesis of ISA16L′

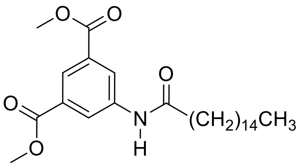

0.66 M palmitoyl chloride **2** in THF (25 mL, 16.5 mmol) was added slowly, dropwise to 5-amino-1,3-dimethyl isophthalate (3.3 g, 15.7 mmol), triethylamine (3.4 mL, 24.7 mmol), and dry THF (90 mL) at 0°C. The reaction mixture was then slowly allowed to warm to 23°C and stirred for at least 1 h. Deionized water (10 mL) was then added, and the THF was removed by rotary evaporation. Diethyl ether (100 mL) and deionized water (50 mL) were added to give a thick suspension which was filtered, washed with deionized water, and dried *in vacuo*. Dimethyl 5-(2′-butyloctanamido)isophthalate **ISA16L′** was obtained as a white solid (7.24 g), which was used for the next saponification step without further

purification. **^{1}H NMR** (600 MHz, $CDCl_3$, ppm) δ 8.41 (s, 2H), 8.38 (s, 1H), 7.81 (1H), 3.91 (s, 6H), 2.40 (t, J = 7.8 Hz, 2H), 1.73 (m, 2H), 1.35 (m, 2H), 1.32-1.20 (m, 22H), 0.87 (t, J = 6.9 Hz, 3H). ^{13}C NMR (150 MHz, $CDCl_3$, ppm) δ 172.1, 166.2, 138.9, 131.5, 126.3, 125.0, 52.7, 37.8, 32.1, 29.9, 29.9, 29.8, 29.8, 29.7, 29.6, 29.6, 29.5.

Synthesis of ISA16L

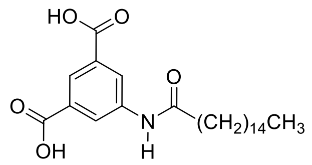

Dimethyl 5-palmitamidoisophthalate **ISA16L′** (7.24 g, 16.2 mmol), KOH (5.76 g, 102.00 mmol), and methanol (200 mL) were heated to reflux. After 1 h, the reaction mixture was cooled to 23°C to give a cloudy white suspension that was acidified with 5 M HCl. The slurry was then filtered, washed with deionized water and dried *in vacuo* to afford **ISA16L** as a white solid (6.45g, 98% based on 5-amino-1,3-dimethyl isophthalate). **^{1}H NMR** (600 MHz, DMSO-d_6, ppm) δ 13.17 (br s, 2H), 10.28 (s, 1H), 8.44 (m, 6H), 8.12 (s, 1H), 2.30 (t, J = 7.2 Hz, 2H), 1.56 (m, 2H), 1.30-1.12 (m, 24H), 0.80 (t, J = 6.9 Hz, 3H). ^{13}C NMR (150 MHz, $CDCl_3$, ppm) δ 171.7, 166.5, 139.9, 131.6, 124.3, 123.4, 36.4, 31.3, 29.0, 29.0, 29.0, 29.0, 28.9, 28.8, 28.6, 25.0, 22.1, 13.9. **HRMS** (EI, M^-) cald for $C_{24}H_{37}NO_5$ 419.2672 $(M\text{-}H)^-$, 418.2599 $(M\text{-}H)^-$ found.

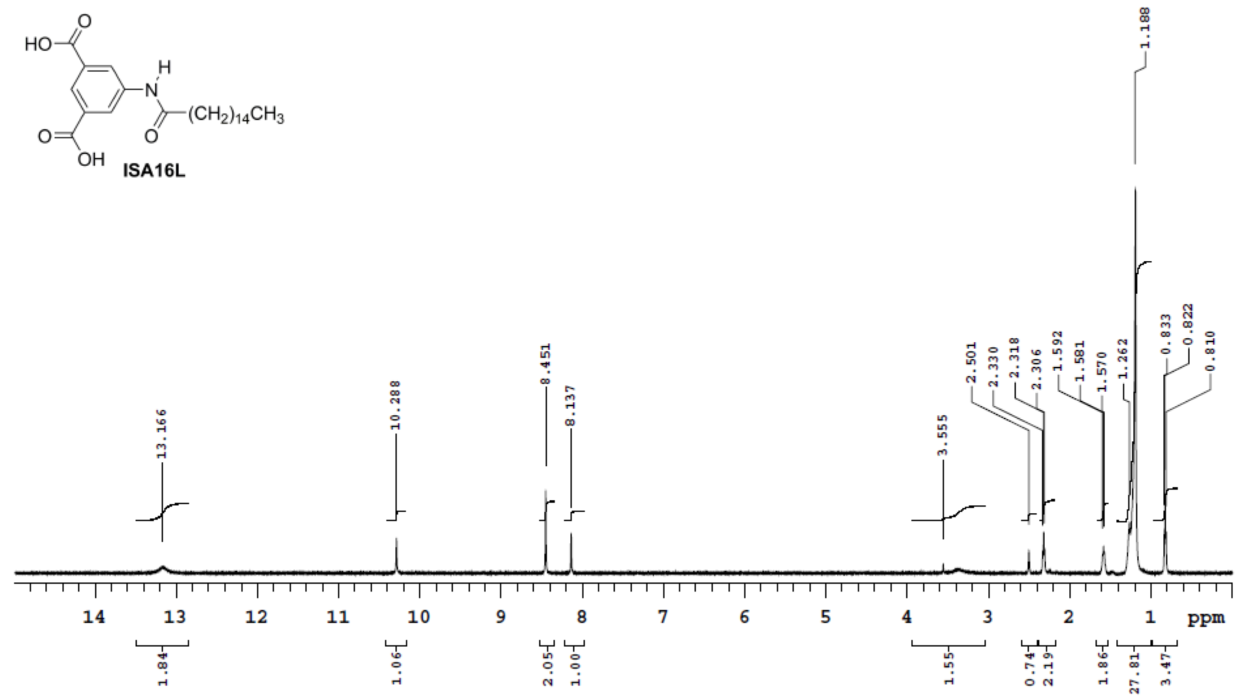

Figure S4. 600 MHz ^{1}H NMR spectrum of **ISA16L** in DMSO-d_6.

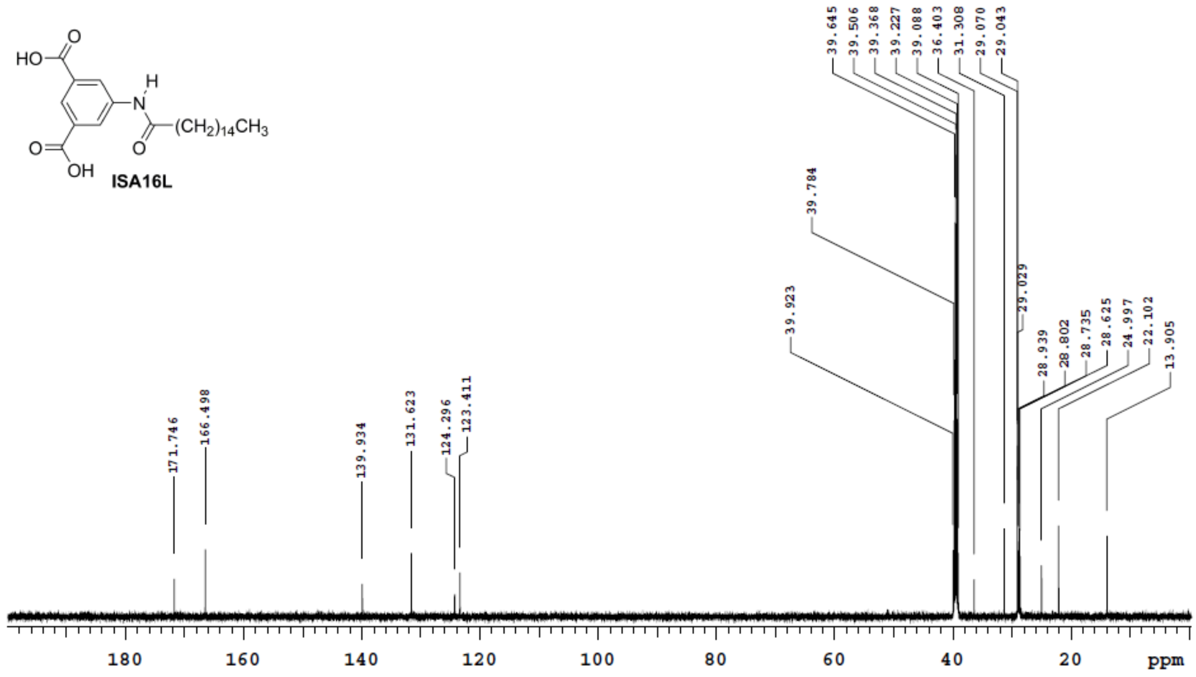

Figure S5. 150 MHz ^{13}C NMR spectrum of **ISA16L** in DMSO-d_6.

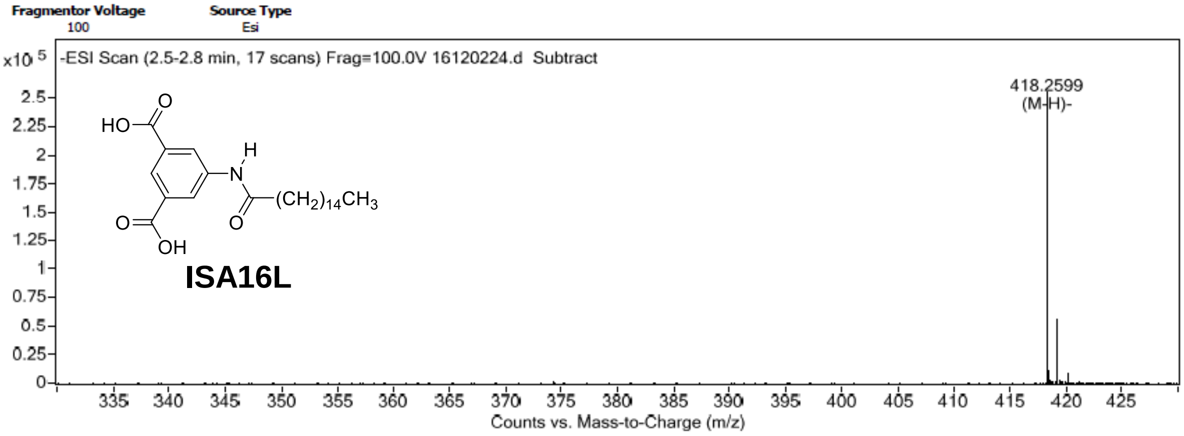

Formula Calculator Results

Formula	Ion Species	Mass	Calc. Mass	m/z	Calc. m/z	Diff (mDa)	Diff(ppm)	DBE	Ion	Score
C24 H37 N O5	C24 H36 N O5	419.2672	419.2672	418.2599	418.2599	-0.02	-0.04	7	(M-H)-	87.72

Figure S6. High Resolution Mass Spectrum of **ISA16L**.

Synthesis of ISA12′

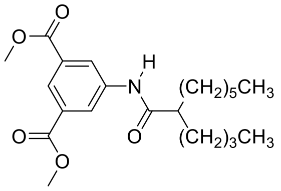

Oxalyl chloride (7.0 mL, 80.2 mmol) was added slowly, dropwise to a solution of 2-butyl octanoic acid **3** (11.4 g, 57.1 mmol), DMF (0.3 mL, 3.9 mmol), and dry THF (200 mL) at 0°C. The reaction was then slowly warmed to room temperature over 2 h before removing the solvent by rotary evaporation. After drying *in vacuo*, the crude 2-butyloctanoic acid chloride **3** was suspended in dry THF (50 mL) and slowly added dropwise to a solution of 5-amino-1,3-

dimethyl isophthalate (11.1 g, 53.0 mmol), triethylamine (11.0 mL, 78.9 mmol), and dry THF (250 mL) at 0°C. The reaction was then allowed to warm slowly to room temperature and stir overnight. After quenching the reaction with water, THF was removed by rotary evaporation. The crude product residue was then dissolved in diethyl ether (200 mL) and was washed successively with saturated sodium bicarbonate (60 mL), deionized water (60 mL), and brine (60 mL). The ether layer was dried over sodium sulfate, filtered, and concentrated by rotary evaporation to give the dimethyl ester **ISA12′** as an amber oil (21.7 g, 97%). **^{1}H NMR** (600 MHz, $CDCl_3$, ppm) δ 8.44 (m, 2H), 8.39 (m, 1H), 7.80 (m, 1H), 3.90 (s, 6H), 2.22 (m, 1H), 1.70 (m, 2H), 1.49 (m, 1H), 1.34-1.18 (m, 12H), 0.90-0.82 (m, 6H). **^{13}C NMR** (150 MHz, $CDCl_3$, ppm) δ 175.3, 166.2, 138.8, 131.5, 126.3, 125.0, 52.6, 49.3, 33.4, 33.1, 31.9, 30.0, 29.6, 27.9, 23.0, 22.8, 14.2, 14.1.

Synthesis of ISA12

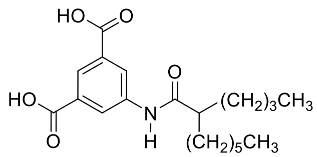

The dimethyl ester **ISA12′** (21.7 g, 55.4 mmol), KOH (85%, 29.56 g, 0.0527 mol), and methanol (200 mL) were heated to reflux overnight. The reaction was then cooled to 23°C to give a red-orange suspension that was acidified with 5 M HCl. The slurry was then filtered, washed with deionized water, and

dried *in vacuo* to afford **ISA12** as a white solid (18.94 g, 94%). **^{1}H NMR** (600 MHz, DMSO-d_6, ppm) δ 13.21 (br s, 2H), 10.25 (s, 1H), 8.47 (m, 2H), 8.14 (s, 1H), 2.34 (m, 1H), 1.55 (m, 2H), 1.36 (m, 2H), 1.30-1.06 (m, 12H), 0.81 (m, 6H). **^{13}C NMR** (150 MHz, DMSO-d_6, ppm) δ 174.8, 166.5, 139. 8, 131.7, 124.4, 123.6, 46.8, 32.6, 32.3, 31.2, 29.2, 28.7, 26.9, 22.2, 22.0, 13.9, 13.9. **HRMS** (EI, M^-) cald for $C_{20}H_{28}NO_5$ 362.1973 $(M-H)^-$, 362.1971 $(M-H)^-$ found.

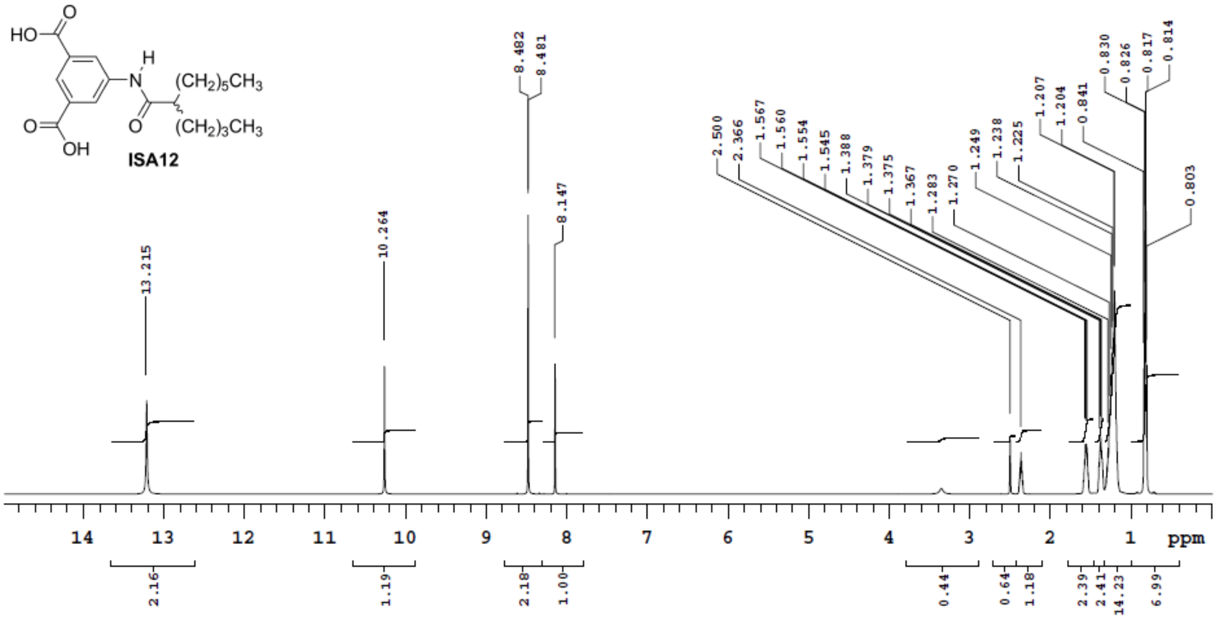

Figure S7. 600 MHz ^{1}H NMR spectrum of **ISA12** in DMSO-d_6.

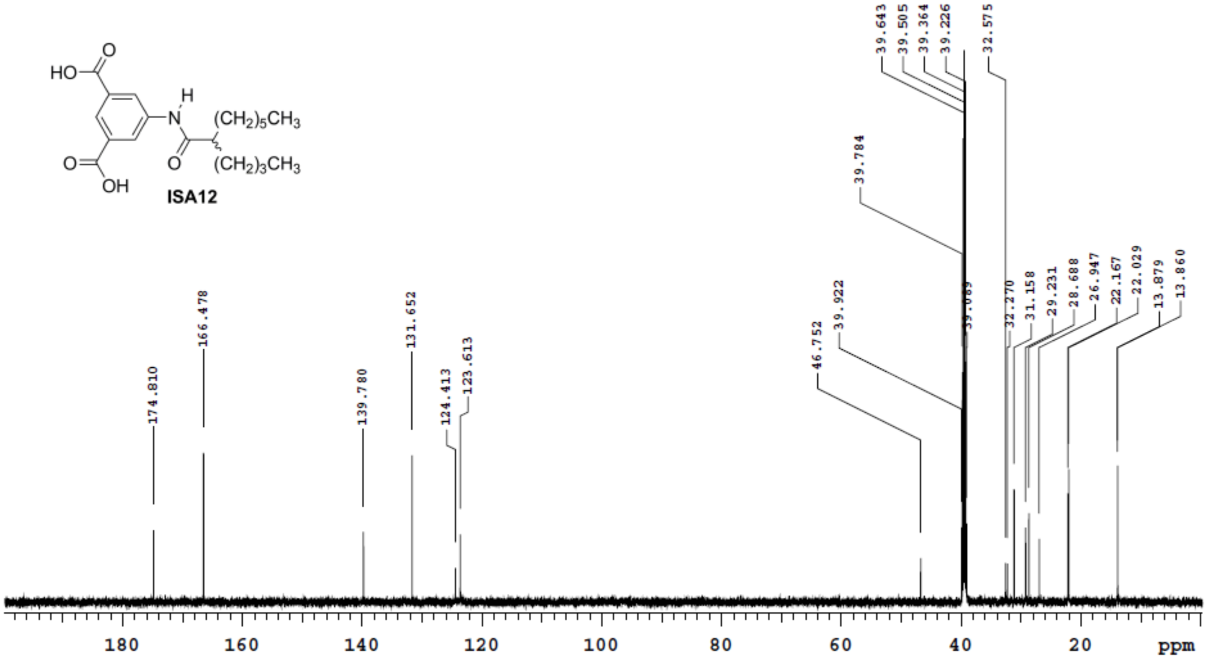

Figure S8. 150 MHz ^{13}C NMR spectrum of **ISA12** in DMSO-d_6.

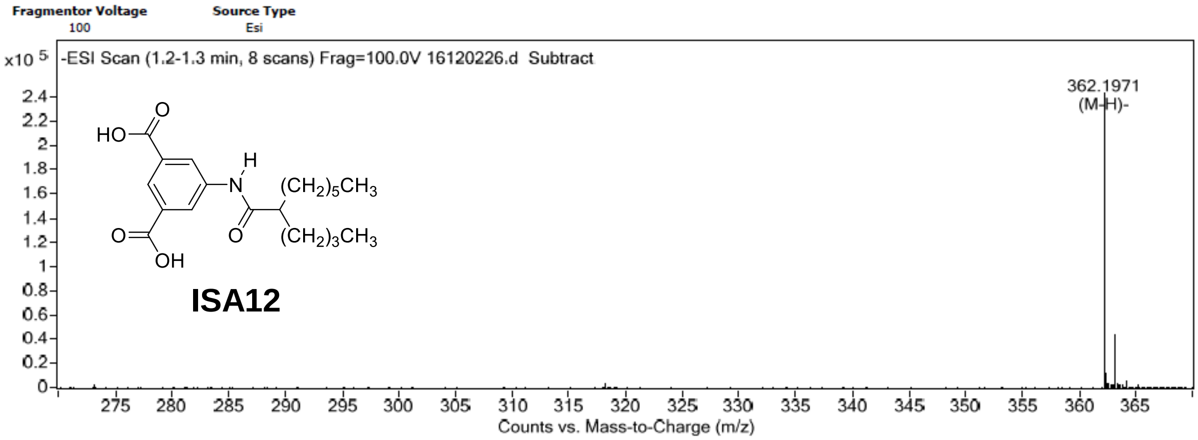

Formula Calculator Results

Formula	Ion Species	Mass	Calc. Mass	m/z	Calc. m/z	Diff (mDa)	Diff(ppm)	DBE	Ion	Score
C20 H29 N O5	C20 H28 N O5	363.2044	363.2046	362.1971	362.1973	0.22	0.61	7	(M-H)-	87.19

Figure S9. High Resolution Mass Spectrum of **ISA12**.

Synthesis of ISA16

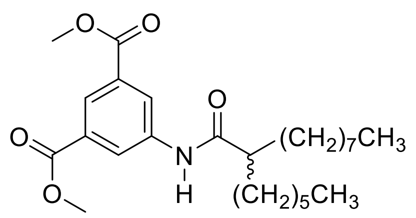

Oxalyl chloride (2.20 mL, 25.6 mmol) was added slowly, dropwise to a solution of 2-hexyldecanoic acid **4** (ISOCARB 16, 3.23 g, 12.6 mmol), DMF (0.100 mL, 1.29 mmol), and dry THF (50 mL) at 0°C. The reaction was then slowly warmed to room temperature over 3 h before removing the solvent by rotary evaporation. After drying *in vacuo*, the crude **4** was suspended in dry THF (70 mL) and slowly added dropwise to a solution of 5-amino-1,3-dimethyl isophthalate (2.65 g, 12.7 mmol), triethylamine (11.0 mL, 78.9 mmol), and dry THF (50 mL) at 0°C. The reaction was then slowly warmed to room temperature and stirred overnight. After quenching the reaction with water, THF was removed by rotary evaporation. The crude product residue was then dissolved in diethyl ether (100 mL) and washed successively with saturated sodium bicarbonate (50 mL), deionized water (50 mL), 5% citric acid (50 mL), and brine (60mL). The ether layer was then dried over sodium sulfate, filtered, concentrated by rotary evaporation and dried *in vacuo* to give the dimethyl ester **ISA16′** as a yellow oil (5.06 g, 90%).

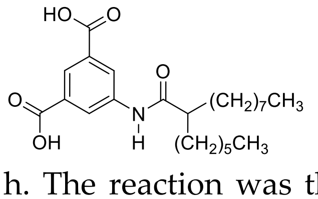

The dimethyl ester **ISA16′** (5.06 g, 11.3 mmol), KOH (85%, 8.39 g, 0.127 mmol), and methanol (200 mL) were heated to reflux for 2 h. The reaction was then cooled to 23°C and acidified with 5 M HCl to give a white precipitate, which coalesced into a gummy mass over 48 h. The supernatant was decanted

away and the gummy mass was dissolved in ethyl acetate (100 mL), which was then washed twice with deionized water (50 mL). After removing the ethyl acetate by rotary evaporation, the waxy solid was triturated with hexanes, filtered, and dried *in vacuo* to give **ISA16** as a fluffy white solid (1.30 g, 26%). **^{1}H NMR** (600 MHz, DMSO-d_6, ppm) δ 13.20 (br s, 2H), 10.20 (br s, 1H), 8.46 (m, 2H), 8.14 (m, 1H), 3.33 (s, 1H), 2.35 (m, 1H), 1.56 (m, 2H), 1.38 (m, 2H), 1.27-1.20 (m, 20H), 0.83-0.80 (m, 6H). **^{13}C NMR** (150 MHz, DMSO-d_6, ppm) δ 174.8, 166.4, 139.7, 131.6, 124.4, 123.6, 46.7, 32.5, 32.5, 31.2, 31.1, 28.9, 28.8, 28.6, 28.6, 26.9, 26.9, 22.0, 22.0, 13.9. **HRMS** (EI, M$^-$) cald for $C_{24}H_{36}NO_5$ 418.2599 (M-H)$^-$, 418.2594 (M-H)$^-$ found.

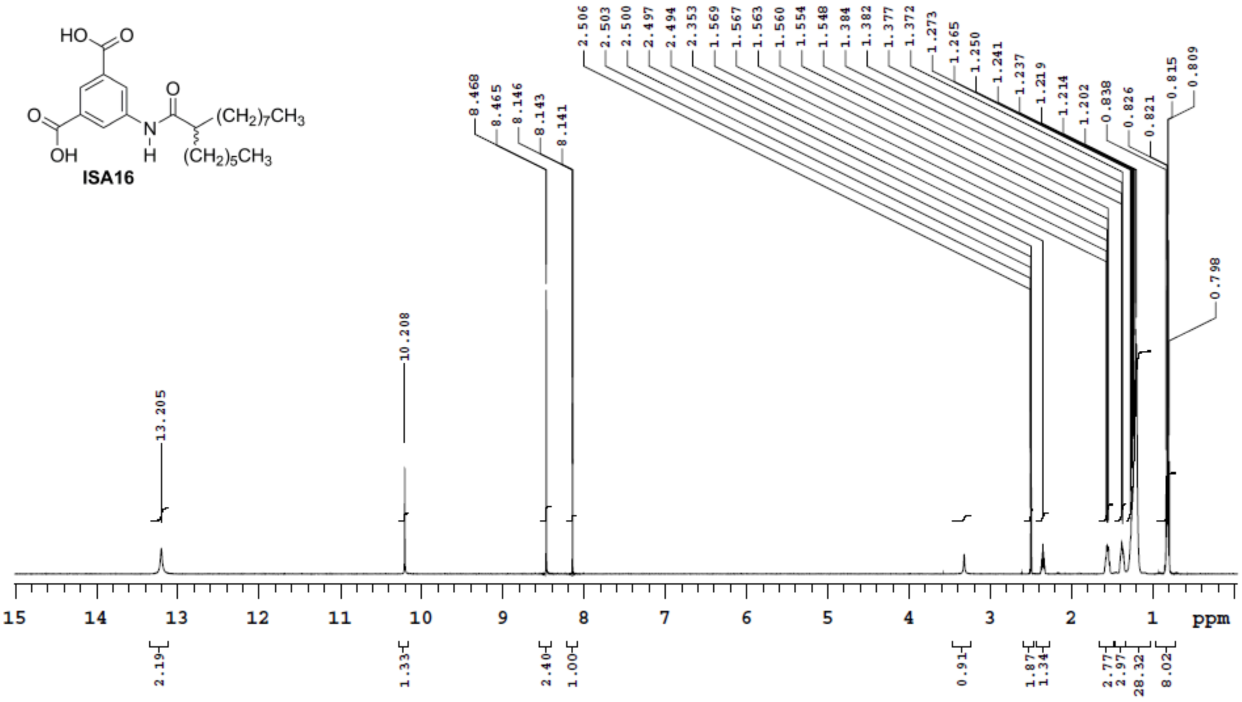

Figure S10. 600 MHz ^{1}H NMR spectrum of **ISA16** in DMSO-d_6.

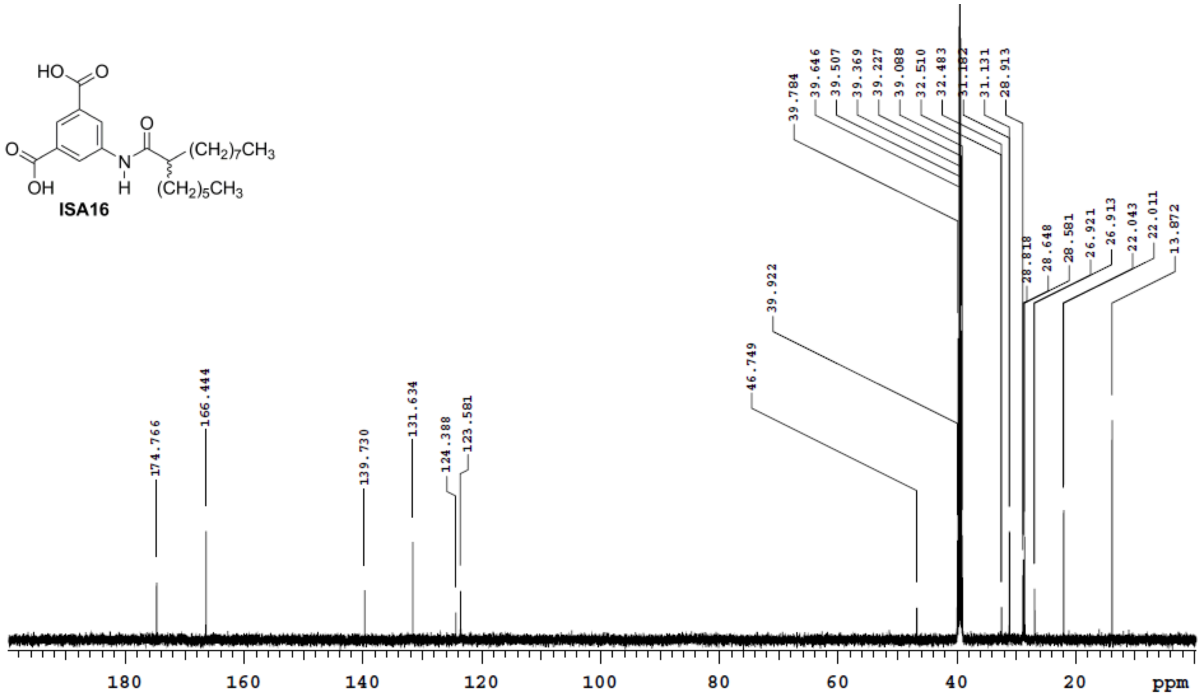

Figure S11. 150 MHz ^{13}C NMR spectrum of **ISA16** in DMSO-d_6.

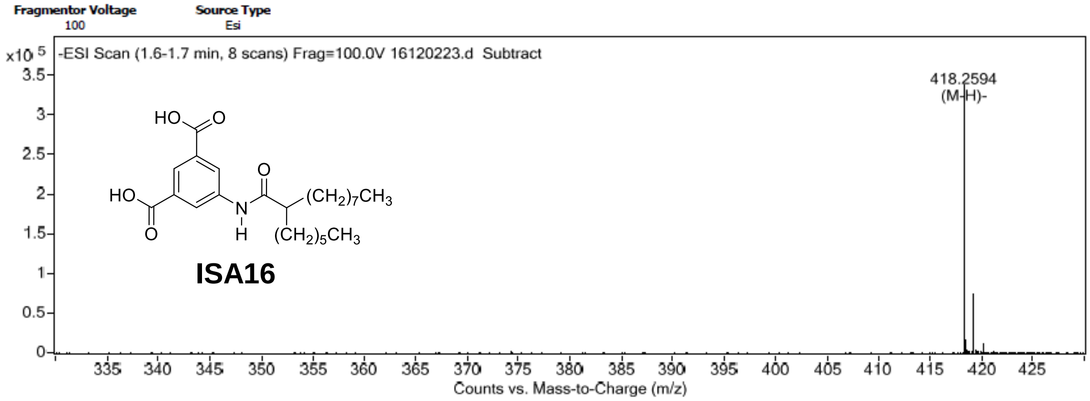

Formula Calculator Results

Formula	Ion Species	Mass	Calc. Mass	m/z	Calc. m/z	Diff (mDa)	Diff(ppm)	DBE	Ion	Score
C24 H37 N O5	C24 H36 N O5	419.2667	419.2672	418.2594	418.2599	0.47	1.12	7	(M-H)-	85.34

Figure S12. High Resolution Mass Spectrum of **ISA16**.

Synthesis of ISA24'

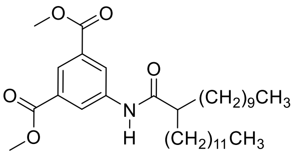

Oxalyl chloride (2.20 mL, 25.6 mmol) was added slowly, dropwise to a solution of 2-decyltetradecanoic acid **5** (ISOCARB 24, 7.65 g, 20.8 mmol), DMF (0.28 mL, 3.62 mmol), and dry THF (100 mL) at 0°C. The reaction was then slowly warmed to room temperature. After stirring overnight at 23°C the solvent was removed by rotary evaporation to afford 2-decyltetradecanoic acid chloride **8** as a pale yellow syrup.

After drying *in vacuo*, the acid chloride **8** was suspended in dry THF (80 mL) and slowly added dropwise to a solution of **DMAISA** (4.40 g, 21.0 mmol), triethylamine (4.4 mL, 31.5 mmol), and dry THF (100 mL) at 0°C. The reaction was then slowly warmed to room temperature and stirred overnight. Deionized water was added and the THF was removed by rotary evaporation. The crude product residue was then dissolved in ethyl

acetate (250 mL) and washed three times with deionized water (100 mL). The ethyl acetate layer was then dried over sodium sulfate, filtered, concentrated by rotary evaporation, and dried *in vacuo*. The dimethyl ester **ISA24'** was obtained as a pale yellow solid (12.56 g), which was used in the next saponification step without further purification. **^{1}H NMR** (600 MHz, DMSO-d_6, ppm) δ 8.42 (m, 2H), 8.41 (m, 1H), 7.40 (s, 1H), 3.92 (s, 6H), 2.18 (m, 1H), 1.68 (m, 2H), 1.50 (m, 2H), 1.40-1.07 (m, 36H), 0.86 (m, 6H). **^{13}C NMR** (150 MHz, DMSO-d_6, ppm) δ 175.1, 166.2, 138.7, 131.6, 126.4, 124.9, 52.7, 49.5, 33.4, 32.1, 32.1, 29.9, 29.9, 29.8, 29.8, 29.7, 29. 6, 29.5, 27.9, 22.9, 22.9, 14.3.

Synthesis of ISA24

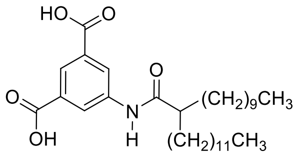

The dimethyl ester **ISA24'** (21.71 g, 55.4 mmol), KOH (4.67 g, 83.2 mmol), and methanol (100mL) were heated to reflux overnight. The reaction was then cooled to 23°C to give a red-orange suspension that was acidified with 5 M HCl. The precipitate was then filtered, washed with deionized water, and dried *in vacuo* to afford **ISA24** as an off-white solid (11.7 g, 100%). **^{1}H NMR** (600 MHz, DMSO-d_6, ppm) δ 10.08 (br s, 1H), 8.35 (m, 2H), 8.18 (m, 1H), 2.37 (m, 1H), 1.55 (m, 2H), 1.34 (m, 2H), 1.42-1.22 (m, 36H), 0.92 (m, 6H). **^{13}C NMR** (150 MHz, DMSO-d_6, ppm) δ 174.3, 166.7,

138.8, 134.7, 124.8, 122.8, 46.5, 32.5, 31.3, 31.3, 29.0, 29.0, 29.0, 28.9, 28.9, 28.7, 26.9, 22.1, 13.9. **HRMS** (EI, M^-) cald for $C_{32}H_{52}NO_5$ 531.3851 $(M\text{-}H)^-$, 530.3853 $(M\text{-}H)^-$ found.

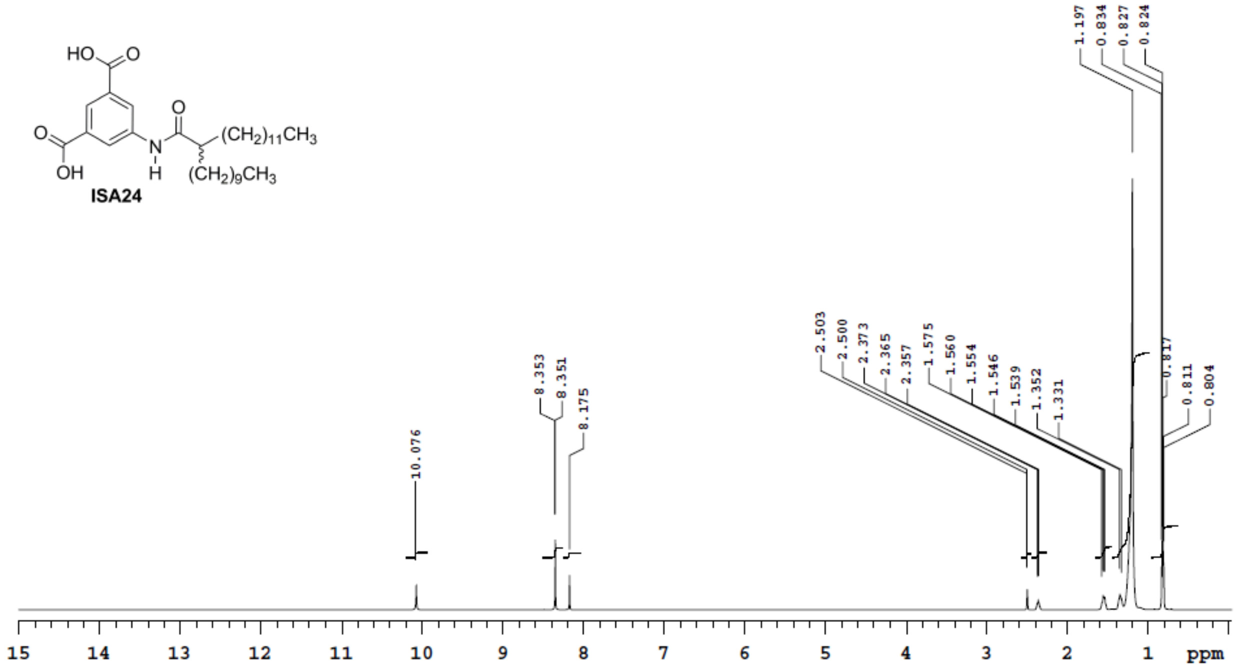

Figure S13. 600 MHz ^{1}H NMR spectrum of **ISA24** in DMSO-d_6.

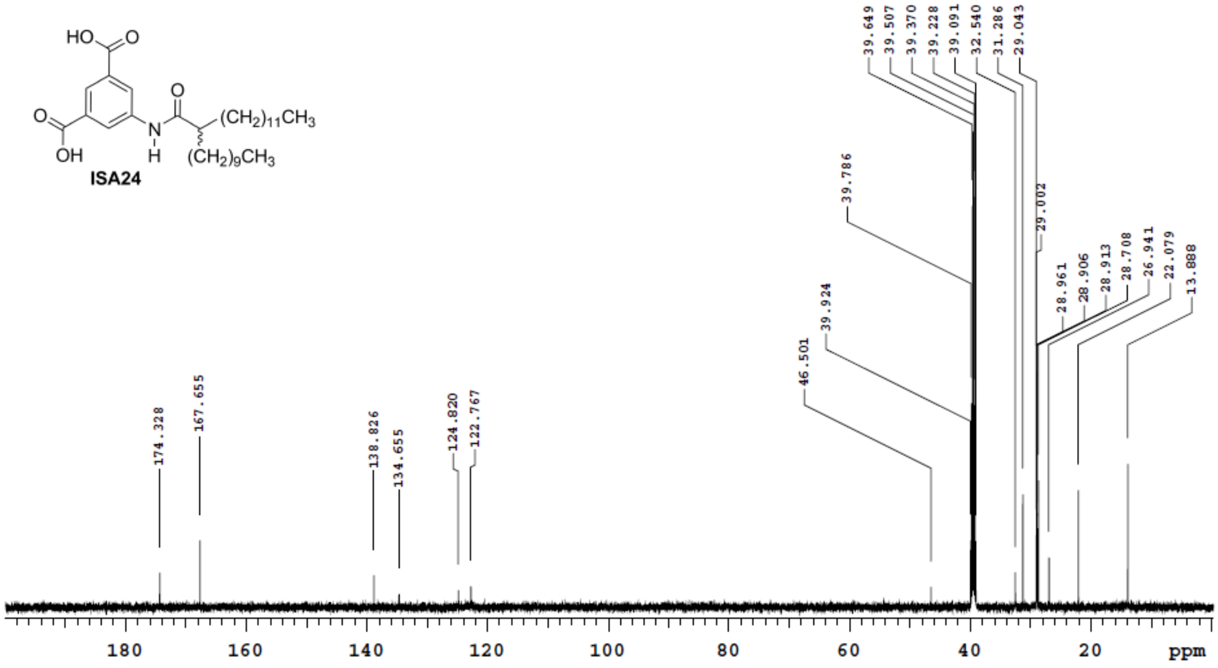

Figure S14. 150 MHz ^{13}C NMR spectrum of **ISA24** in DMSO-d_6.

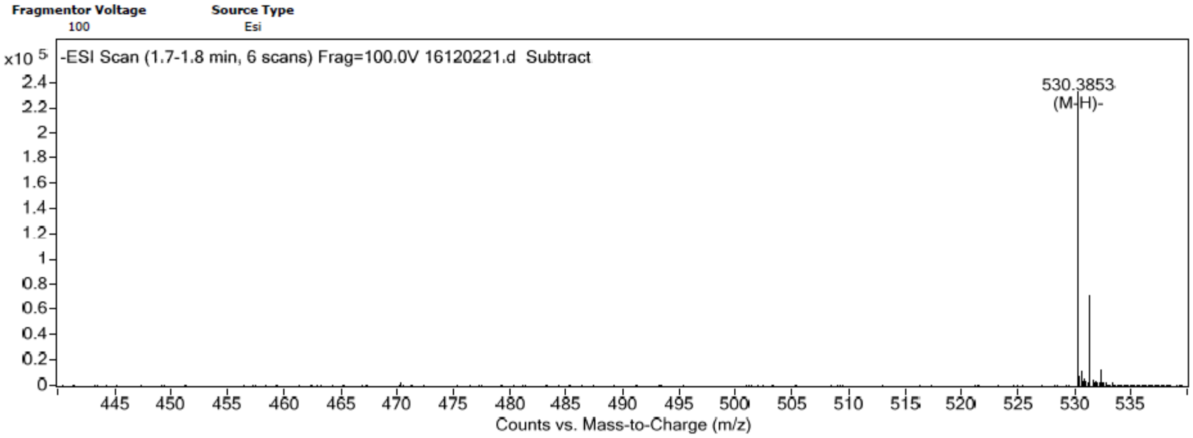

Formula Calculator Results

Formula	Ion Species	Mass	Calc. Mass	m/z	Calc. m/z	Diff (mDa)	Diff(ppm)	DBE	Ion	Score
C32 H53 N O5	C32 H52 N O5	531.3926	531.3924	530.3853	530.3851	-0.24	-0.46	7	(M-H)-	88.72

Figure S15. High Resolution Mass Spectrum of **ISA24**.

Gelation Behavior

Table S1. Appearances of ISA gelators in different liquids and CGC values (wt %) of the gels

Liquid	ISA4	ISA16L	ISA12	ISA16	ISA24
Water	I	I	I	I	I
Dimethyl sulfoxide	S	S	S	S	S
Ethylene Glycol	S	OG (0.5)	S	OG (<1.0)	TG (2.4)
Methanol	S	S	S	S	S
Ethanol	-	OG (<10)	OG (20)	-	OG (<10.6)
1-Hexanol	-	-	-	-	OG (<11.4)
2-Ethyl Hexanol	-	S	S	S	TG (<1.0)
Ethyl Acetate	-	P	S	S	OG (<10)
n-Butyl Acetate	-	-	OG (<0.5)	-	OG (<2.3)
Acetonitrile	-	P	P	P	P
Acetone	S	OG (<10)	OG (10)	P	I
Tetrahydrofuran	S	S	S	S	OG (<10.0)
1,2-Dimethoxyethane	-	I	S	S	CG (0.3)
Chloroform	I	I	I	I	CG (0.5)
Cyclohexane	-	P	I	I	CG (0.7)
Methyl cyclohexane	-	-	-	-	CG (1.0)
Decalin	-	TG (0.6)*	TG (<0.1)*	TG (<0.1)*	CG (0.2)
Benzene	-	-	PG	TG (<1.0)	CG (<0.2)
Toluene	I	TG (<1.0)*	TG (<1.0)*	TG* (0.7)	CG (0.7)
Xylenes	I	TG (<0.4)*	TG (<0.5)*	CG (<0.1)*	CG (0.2)
Mesitylene	-	TG (1.2)*	TG (<2.0)*	TG (<2.0)*	CG(<0.1)
Styrene	-	TG (<1.0)*	TG (<1.0)*	TG (<1.9)*	CG (<1)
Chlorobenzene		I	TG (<1.0)*	OG (<2.0)*	CG (<0.2)
Benzyl benzoate	-	P	TG (<1.0)	TG (<1.0)	TG (<1.0)
Aniline	-	OG (<2.5)	TG (<2.5)	TG (<2.2)	TG (<1.9)
Hexanes	I	I	I	I	I
Dodecane	-	CG (1-2.1)	I	PG	CG (<1.0)
Hexadecane	-	CG (0.6)	I	I	CG (2.3)
2,2,4-trimethylpentane	-	I	-	-	TG (<1.0)*
Kerosene	-	CG (0.4-0.6)*	TG (<0.1)*	-	CG (0.5)

Liquid	ISA4	ISA16L	ISA12	ISA16	ISA24
Paraffin oil	-	TG (<0.1)*	TG (<0.1)*	TG (<0.2)	TG (0.1)
Gasoline	-	-	-	-	CG (0.7)*
Crude oil (Syncrude Sweet)	-	S	PG	PG	TG (0.5-0.8)
Vacuum Pump Oil	-	-	-	-	CG (<1.2)
Olive oil	-	-	-	-	CG (<1)
Canola oil	-	-	-	-	OG (<1.2)
Soybean oil	-	-	-	-	I

I = insoluble. OG = opaque gel. S = Solution. TG = turbid gel. P = precipitate. CG = clear gel. PG = partial gel. * = gel decomposed to a partial gel or significant syneresis occurred over time.

Hansen Solubility Parameters

Table S2. Hansen solubility parameters (MPa$^{1/2}$) for and gelation tests with various solvents for 5-alkylamido ISA derivatives (1 wt %).

Solvent	δ_p	δ_h	δ_d	$2\delta_d$	ISA12	ISA16	ISA24	ISA16L
1,2-Dimethoxyethane	6.20	6.50	15.50	31.00	S	S	G	S
2-Ethylhexanol	3.30	11.80	15.90	31.60	S	S	G	S
Acetone	10.40	7.00	15.50	31.00	S*	S*	S	S
Acetonitrile	18.00	6.10	15.30	30.60	I	I	I	I
Benzyl benzoate	5.10	5.20	20.00	40.00	G	G	I*	S
Chloroform	3.10	5.70	17.80	35.60	I	I	G	I
Cyclohexane	0.00	0.20	16.08	33.60	I	I	G	I
Decalin	0.00	0.00	17.80	36.80	G	G	G	G
DMSO	16.40	10.20	18.40	36.80	S	S	S	S
Dodecane	0.00	0.00	16.00	32.00	I	PG	G	S*
Ethyl Acetate	5.30	7.20	15.80	31.60	S	S	I*	I
Ethylene Glycol	11.00	26.00	17.00	34.00	S	G	G	G
Methanol	12.30	22.30	14.70	29.40	S	S	S	S
THF	5.70	8.00	16.80	33.60	S	S	S*	S
Toluene	1.40	2.00	18.00	36.00	G	G	G	G
Water	16.00	42.30	15.50	31.00	I	I	I	I

Xylenes	1.65	2.70	17.90	35.80	G	G	G	G
1,2-Dichloroethane	7.40	4.10	18.00	36.00	-	-	I	-
2,2,4-Trimethylpentane	0.00	0.00	14.10	28.20	-	-	S	I
2-Butanone	9.00	5.10	16.00	32.00	S	-	-	-
Aniline	5.80	11.20	20.10	40.20	-*	-*	S*	-*
Benzene	0.00	2.00	18.40	36.80	-	G	PG*	-
Chlorobenzene	4.30	2.00	19.00	38.00	G*	-*	S*	S
Ethanol	8.80	19.4	15.80	31.60	S*	-	S*	-*
Hexadecane	0.00	0.00	16.30	32.60	-	I	I*	G
Hexanes	0.00	0.00	14.90	29.80	-	-	I	I
Mesitylene	0.60	0.60	18.00	36.00	-*	-*	S*	G
Pyridine	8.80	5.90	19.0	38.00	S	-	S	-
Styrene	1.00	4.10	18.60	37.20	G	-*	G	G

Experiments listed in black were used for gel sphere fitting. Experiments listed in red are additional data at 1 wt %. *Denotes that gels were formed at higher gelator concentrations.

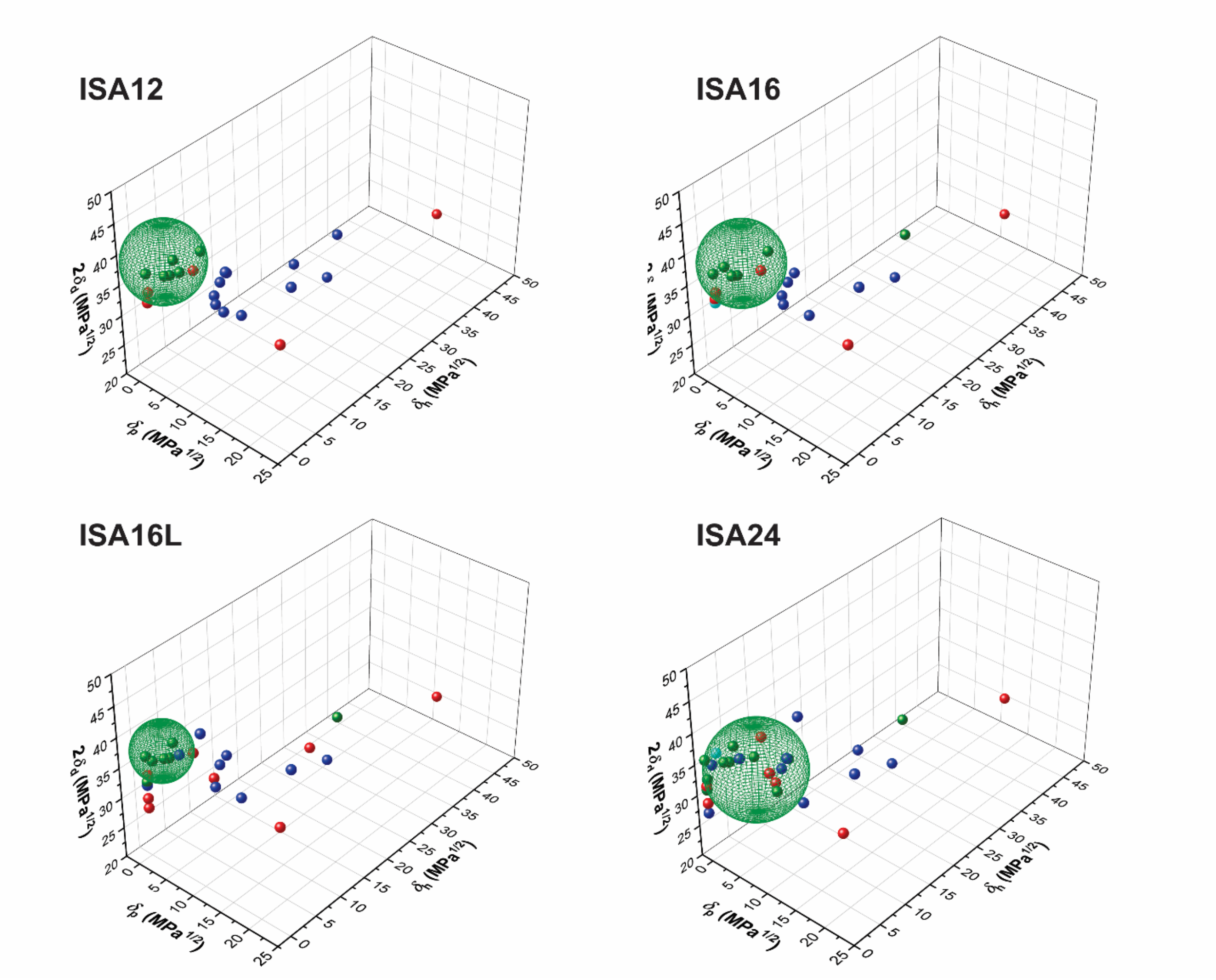

Figure S16. Solubility data for organogelators **ISA12**, **ISA16**, **ISA16L**, and **ISA24** represented in Hansen space. Green sphere is the gelation region. Green, blue, and red data points indicate gels, soluble, and insoluble/precipitates, respectively.

Table S3. Hansen coordinates and radii (MPa$^{1/2}$) for the gel spheres for 5-alkylated ISA derivatives.

	δ_p	δ_h	δ_d	R	Fit
**ISA12**	2.51	1.01	19.72	5.73	1.00
**ISA16**	3.48	1.90	19.57	5.88	1.00
**ISA16L**	1.93	1.18	18.98	4.27	1.00
**ISA24**	5.98	3.20	18.20	6.76	0.95

Gel Characterization

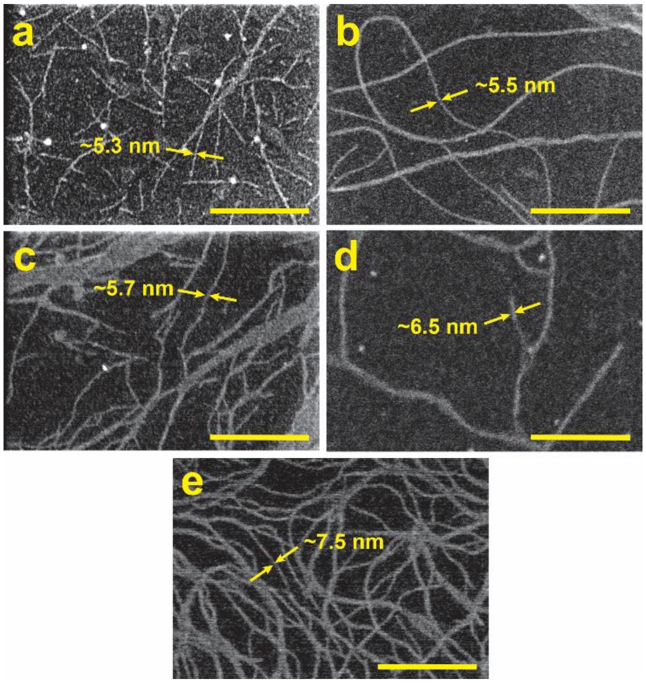

Figure S17. SEM images of nanofibrils deposited from 5-alkylated ISA derivatives. (a) **ISA4** in 1 : 1 xylenes : THF (1 mg/mL), (b) **ISA12** in 19 : 1 xylenes : THF (1 mg/mL), (c) **ISA16** in xylenes (1 mg/mL), (d) **ISA16L** in 19 : 1 xylenes : THF (0.45 mg/mL), (e) **ISA24** in $CHCl_3$ (0.1 mg/mL). The yellow arrows indicate individual nanofibrils. The cosolvent THF was required for all compounds except for **ISA24** to promote better solubility in xylenes.

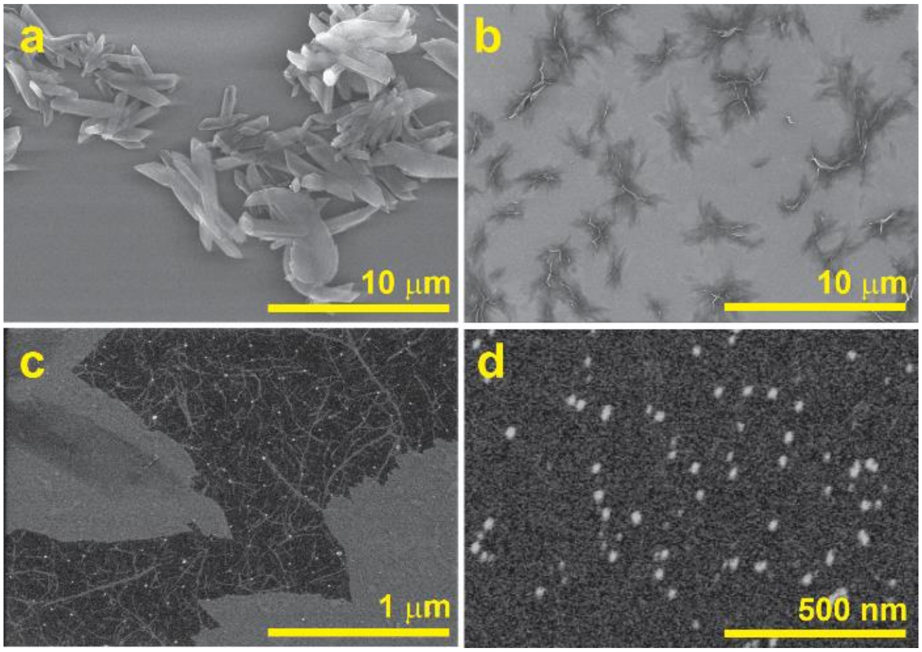

Figure S18. SEM images of self-assembled nanostructures of **ISA4** deposited from solutions. (a) THF : Xylenes (1 : 9, 1 mg/mL). (b) and (c) THF : Xylenes (1 : 1, 1 mg/mL). (d) THF solution (0.1 mg/mL).

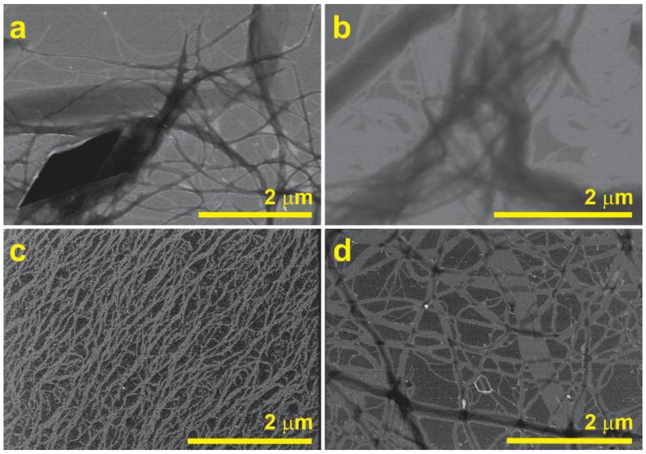

Figure S19. SEM images of xerogels, SAFINs, self-assembled nanofibers of 5-alkylamido ISAs deposited from toluene solutions (0.1 mg/mL). (a) **ISA12**. (b) **ISA16**. (c) **ISA24**. (d) **ISA16L**.

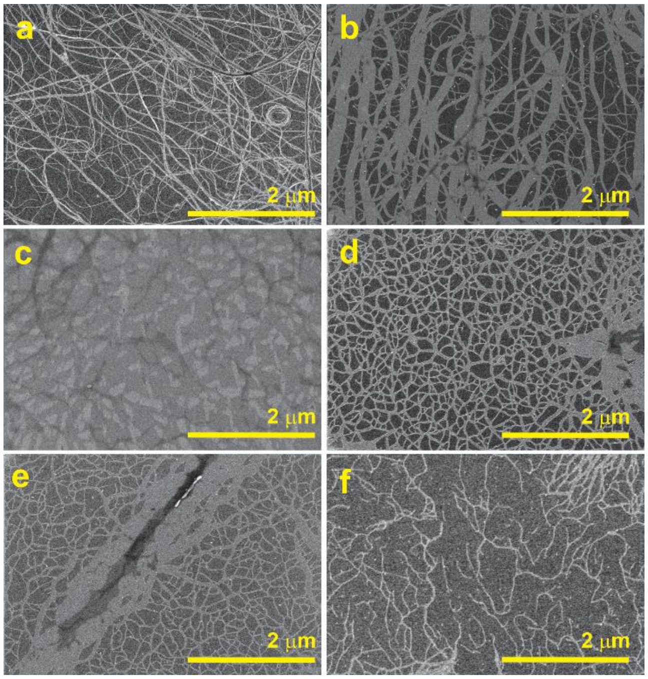

Figure S20. SEM images of SAFINs of **ISA12**, **ISA16**, and **ISA16L** deposited from xylenes : THF (19:1) solutions. (a) **ISA12**, 1 mg/mL. (b) **ISA16** (1 mg/mL). (c) **ISA16L** (1 mg/mL). (d) **ISA16L** (0.4 mg/mL). (e) **ISA16L** (0.1 mg/mL). (f) **ISA16L** (0.06 mg/mL).

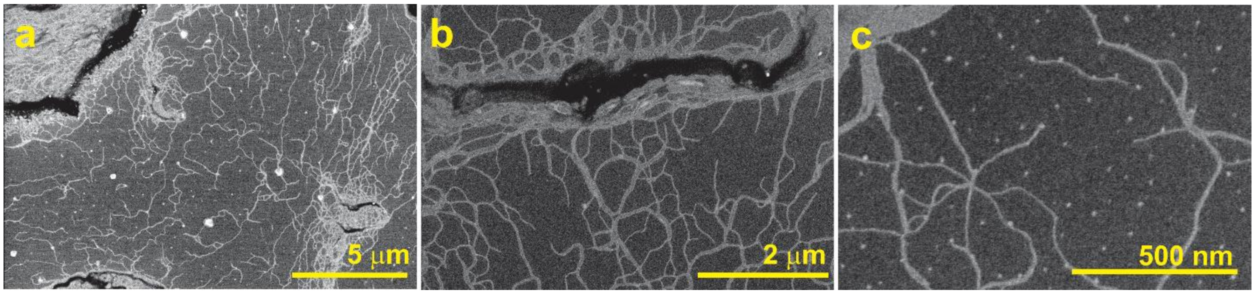

Figure S21. SEM images of self-assembled nanostructures of **ISA24** deposited from cyclohexane. (a) 20 mg/mL, gel film edge. (b) 0.2 mg/mL. (c) 0.1 mg/mL.

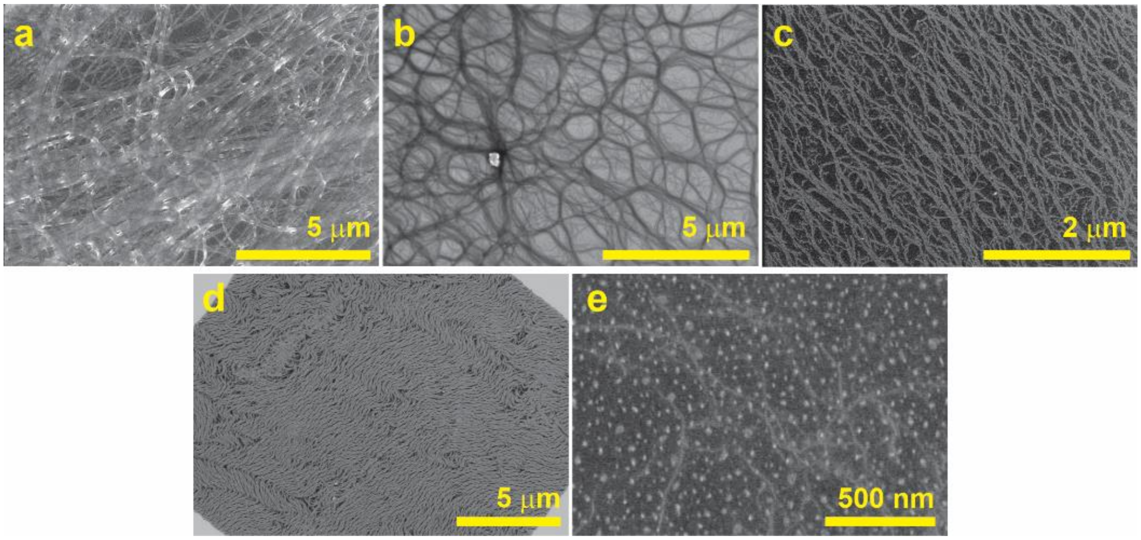

Figure S22. SEM images of self-assembled nanostructures of **ISA24** deposited from toluene. (a) 10 mg/mL. (b) 4 mg/mL. (c) and (d) 0.1 mg/mL. (e) 0.01 mg/mL.

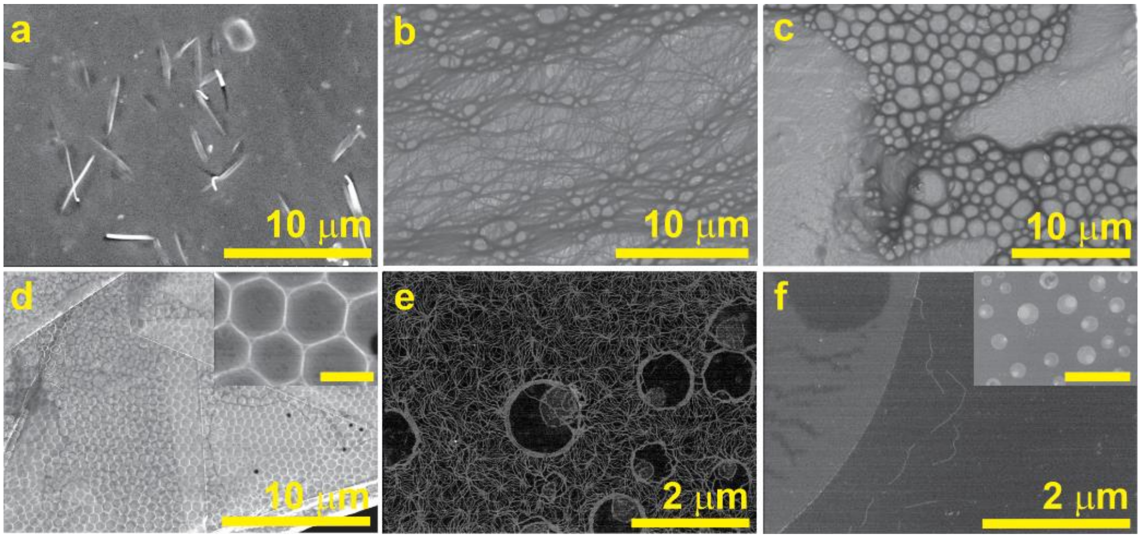

Figure S23. SEM images of self-assembled nanostructures of **ISA24** deposited from $CHCl_3$. (a) 10 mg/mL. (b) 1 mg/mL. (c) - (e) 0.1 mg/mL, dense and sparse regions. (e) 0.065 mg/mL.

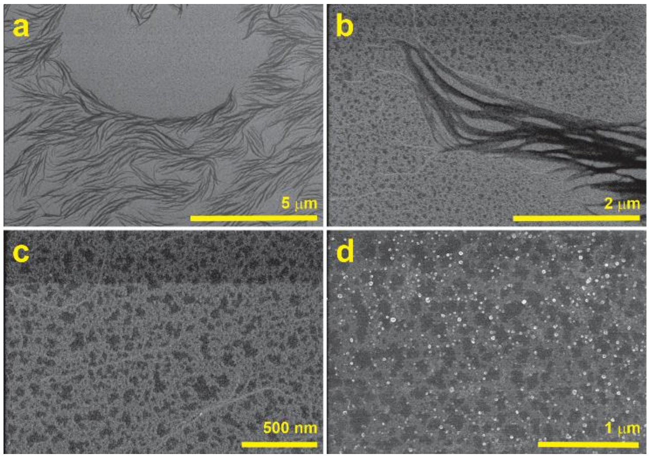

Figure S24. SEM images of self-assembled nanostructures of **ISA24** deposited from THF. (a) – (c) 1 mg/mL. (d) 0.1 mg/mL.

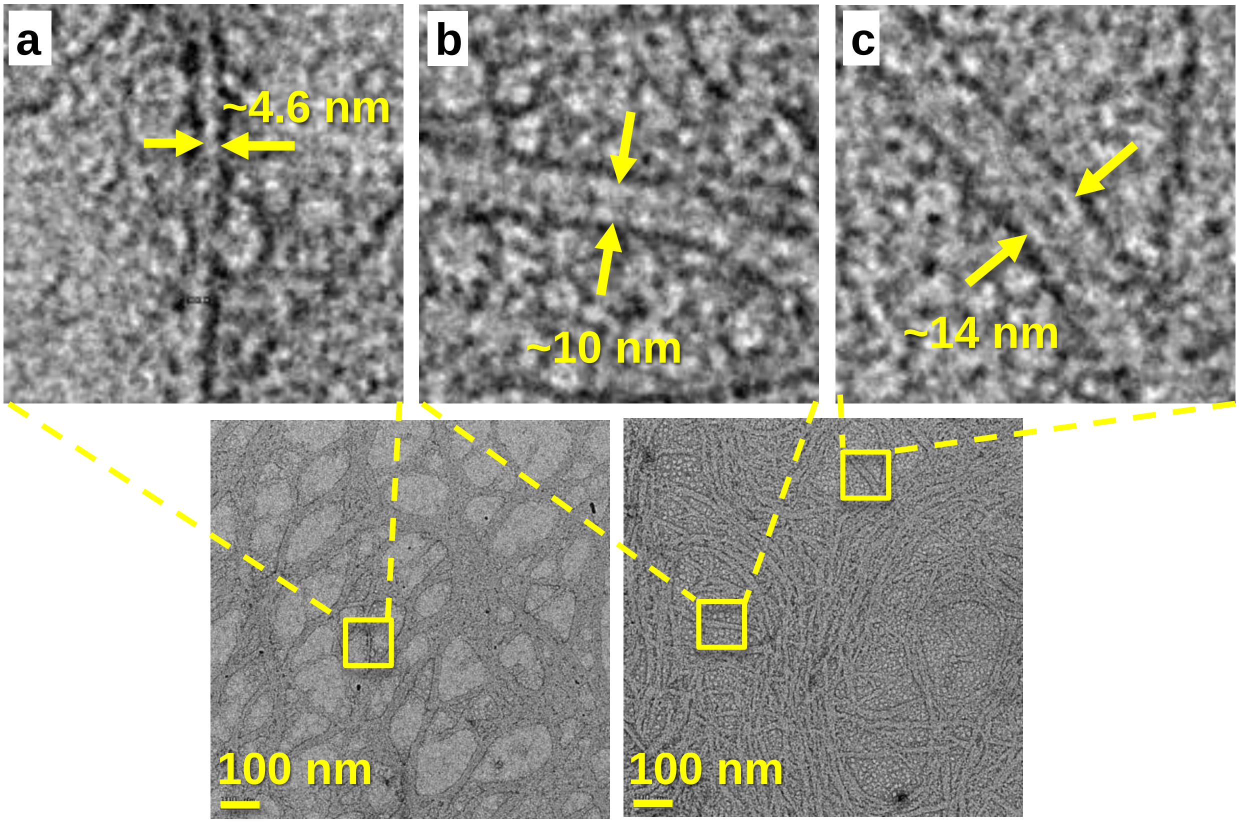

Figure S25. HR-TEM images of nanofibrils from **ISA24** formed in cyclohexane (0.02 wt %). (a) Single nanofibril. (b) 2 nanofibril bundle. (c) 3 nanofibril bundle.

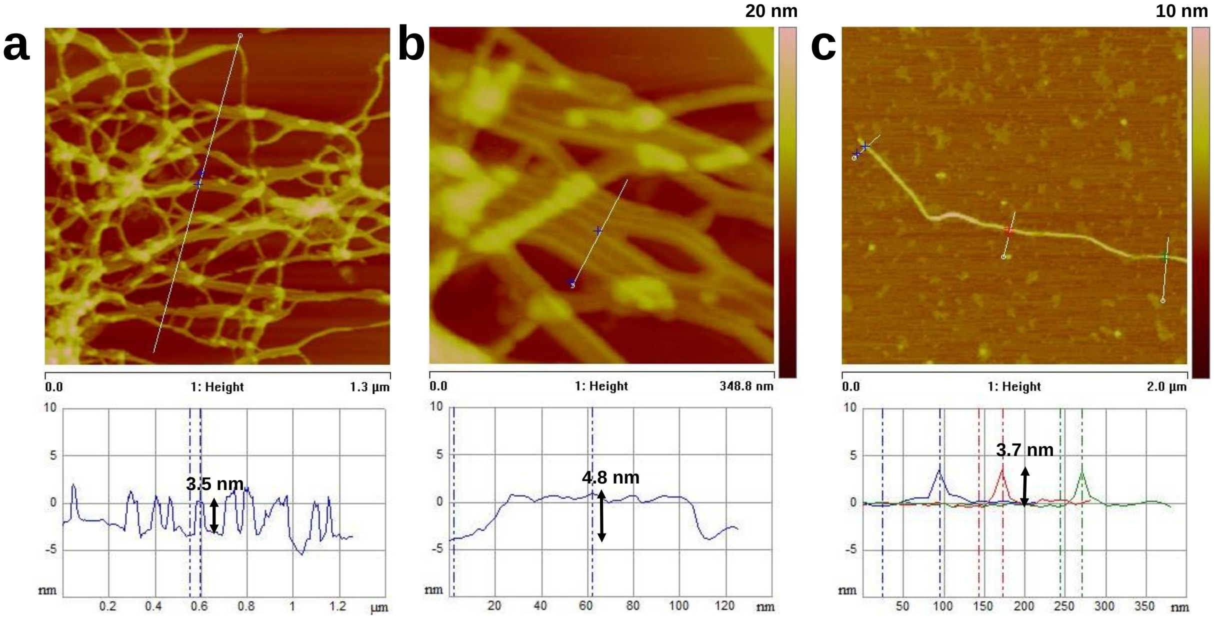

Figure S26. AFM height images of nanofibrils formed by **ISA24** in cyclohexane (0.02 wt %) on freshly cleaved mica. Cross sectional analysis across (a) several bundled nanofibrils (1.3 µm × 1.3 µm, vertical scale bar represents 0-20 nm), (b) a large bundle of nanofibrils (348.8 nm × 348.8 nm, vertical scale bar represents 0-20 nm), and (c) an individual primary nanofibril (2.0 µm × 2.0 µm, vertical scale bar represents 0-10 nm).

Table S4. Measured nanofiber widths and cyclic hexamer diameters from computer models (Figure S20) for alkylamido ISA derivatives.

**Alkylamido ISA**	**Solvent**	**Smallest Nanofiber Width (nm)***	**Cyclic Hexamer Diameter (nm)**
**ISA4**	Xylenes:THF (1:1)	~5	3.6
**ISA12**	Xylenes:THF (19:1)	~7	4.1
**ISA16**	Xylenes:THF (9:1)	~8	4.5
**ISA24**	Toluene	~17	5.2
	$CHCl_3$	~9	-
	Hexanes	~22	-
	Cyclohexane	7-10	-

**ISA16L**	Xylenes	~9	6.4

*Measured from SEM images using ImageJ. The error was determined to be ±10%.

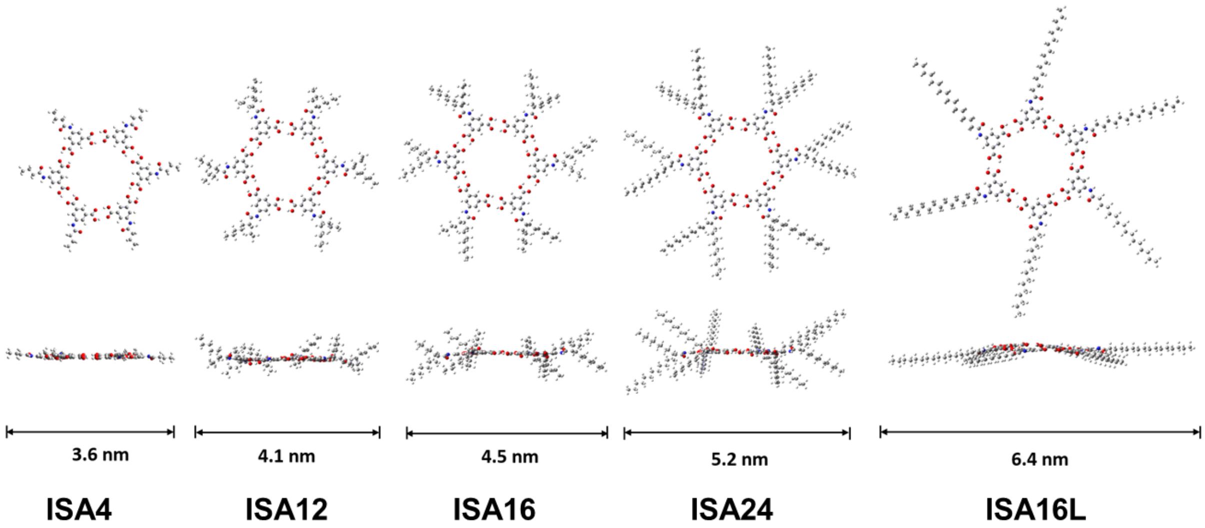

Figure S27. Ball-and-stick computer models of cyclic hexamers from 5-alkylamido ISAs and their approximate maximum diameters. O = red. N = blue. C = grey. H = white.

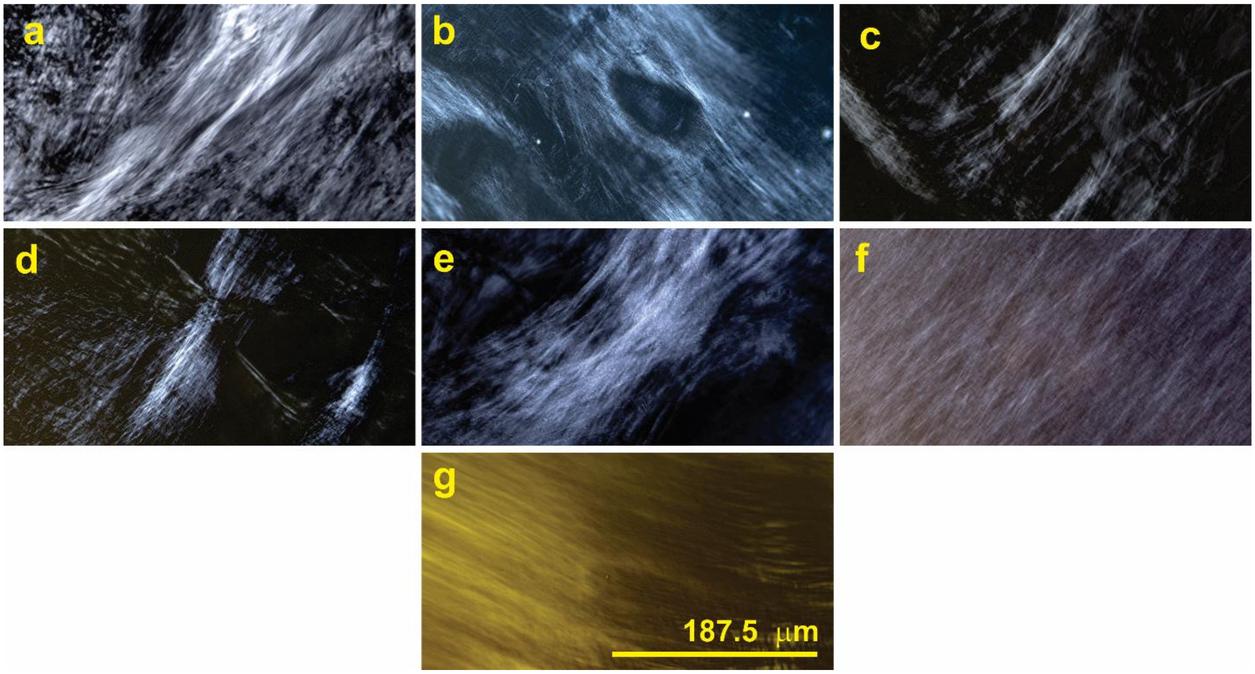

Figure S28. POM images of gels of **ISA24** with different solvents. (a) $CHCl_3$ (1 wt %). (b) Benzene (0.5 wt %). (c) Xylenes (1 wt %). (d) Decalin (1 wt %). (e) Dodecane (1.4 wt %). (f) Paraffin oil (0.3 wt %). (g) Crude oil (1 wt %).

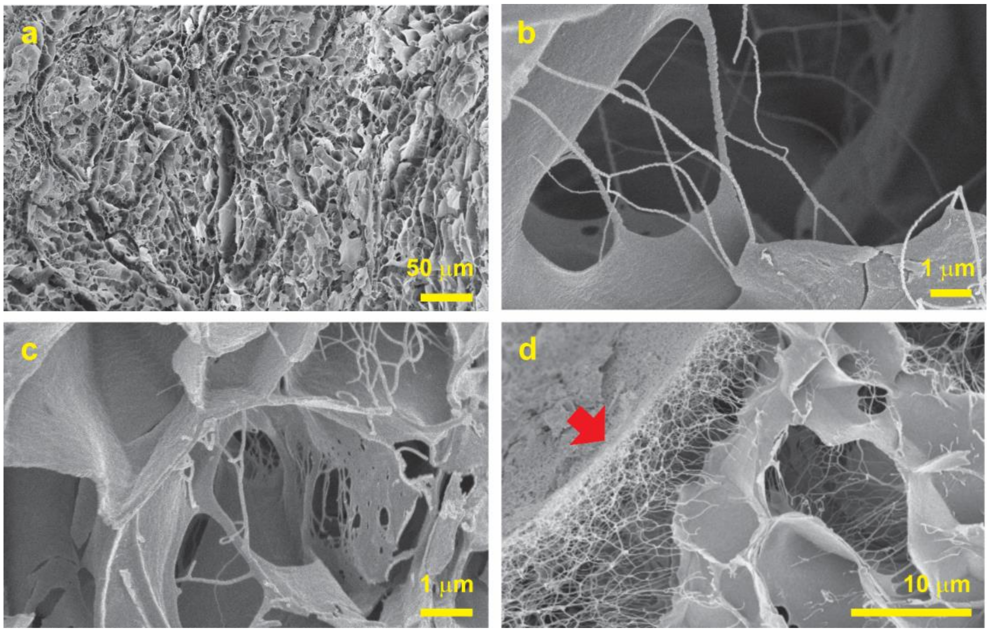

Figure S29. Cryo-SEM images of **ISA24** from cyclohexane (2 wt %). The red arrow in (d) points to a network of nanofibers.

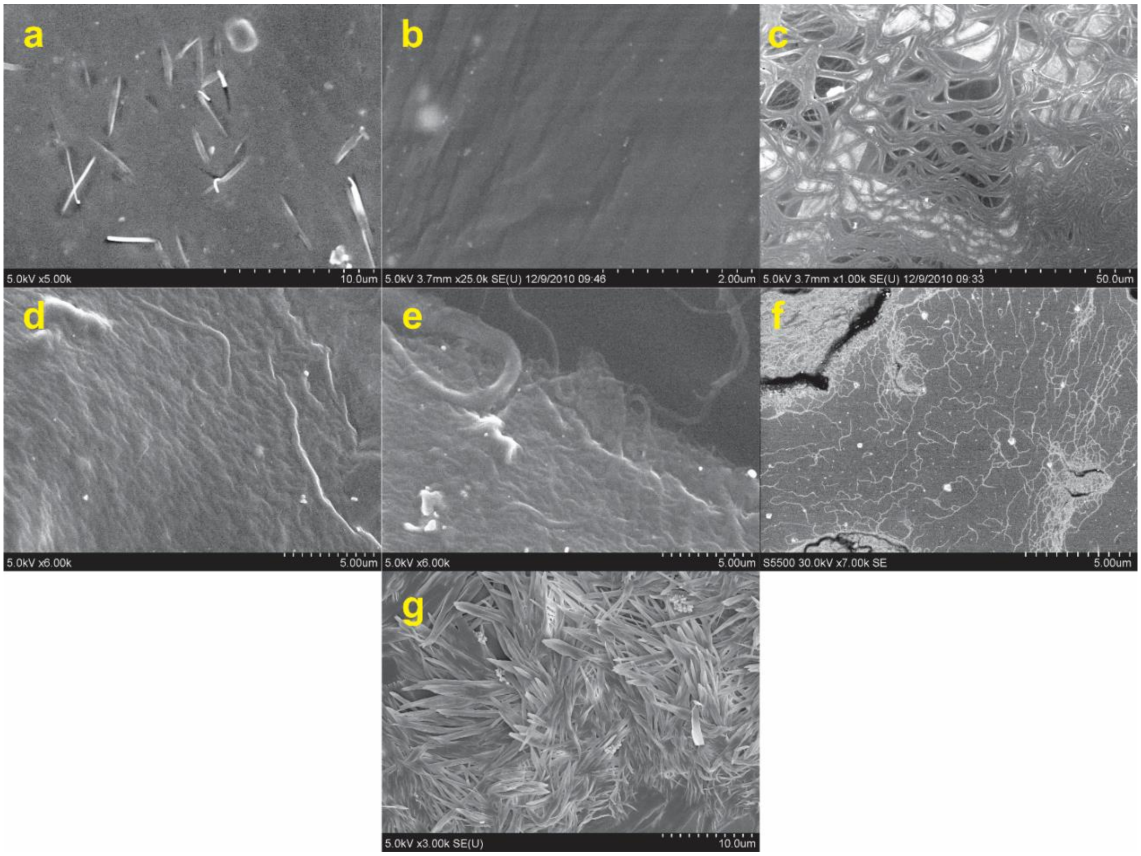

Figure S30. FE-SEM images of xerogels of **ISA24** from different solvents. (a) $CHCl_3$ (2 wt %). (b) and (c) dodecane (2 wt %). (d) and (e) Benzene (2 wt %). (f) Cyclohexane (2 wt %). (g) MeOH (10 wt %).

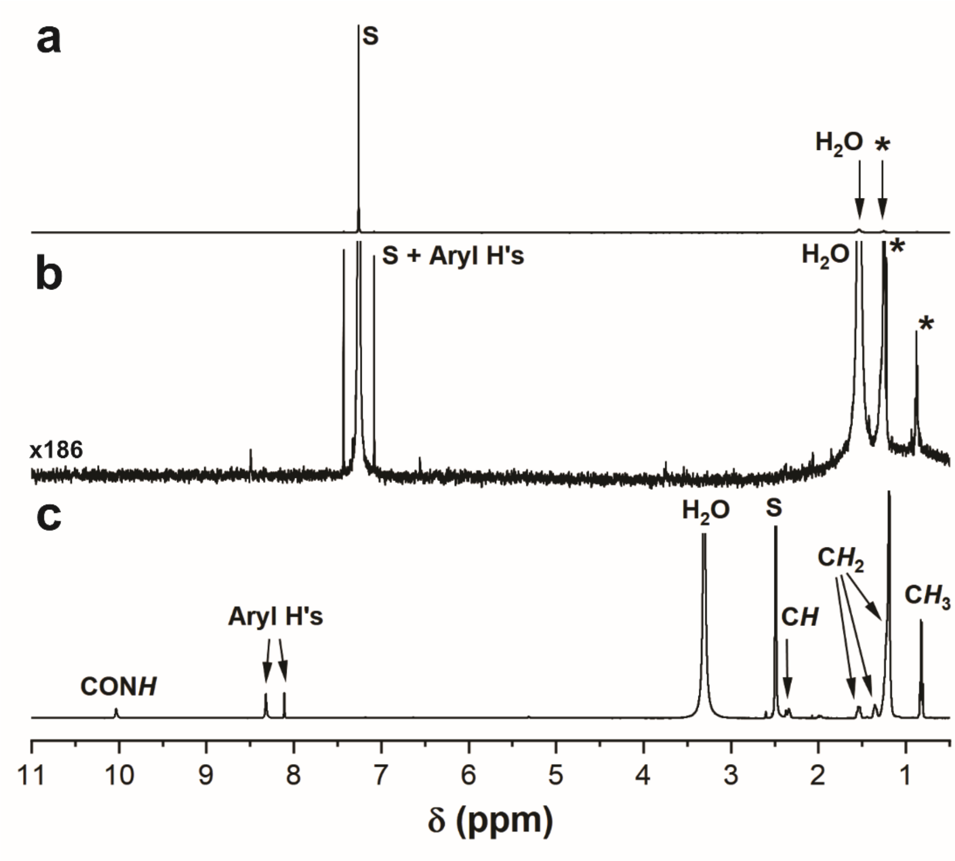

Figure S31. ^{1}H NMR spectra of **ISA24**. (a) $CDCl_3$ (1.9 mM), (b) Same as (a), scaled by × 170. (c) DMSO-d_6 (1.9 mM). S = residual solvent. * = trace "grease" signals. The aryl H's in $CDCl_3$ overlap with the residual solvent signal.

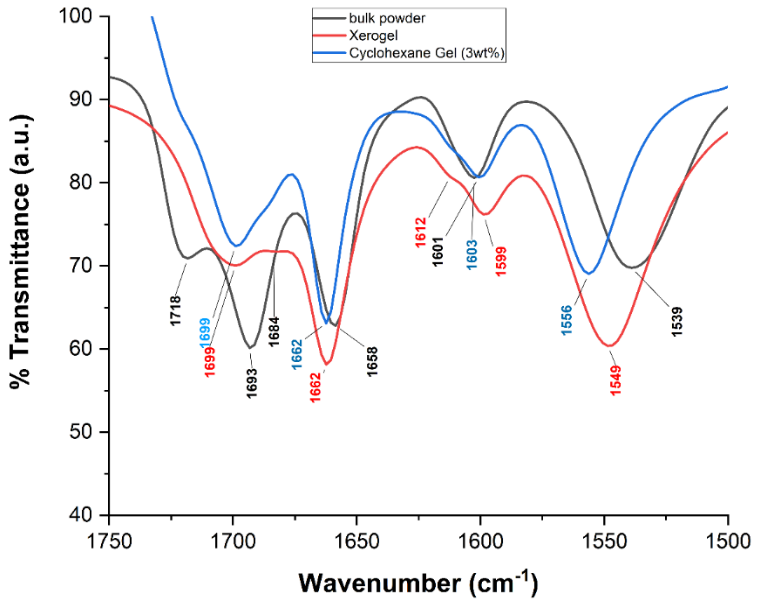

Figure S32. Partial ATR-FTIR spectra of **ISA24**. (a) O-H/N-H stretching region. (b) C=O stretching and N-H bending region. Black trace = bulk powder. Blue trace = organogel with cyclohexane (3 wt %). Red trace = xerogel (from cyclohexane gel).

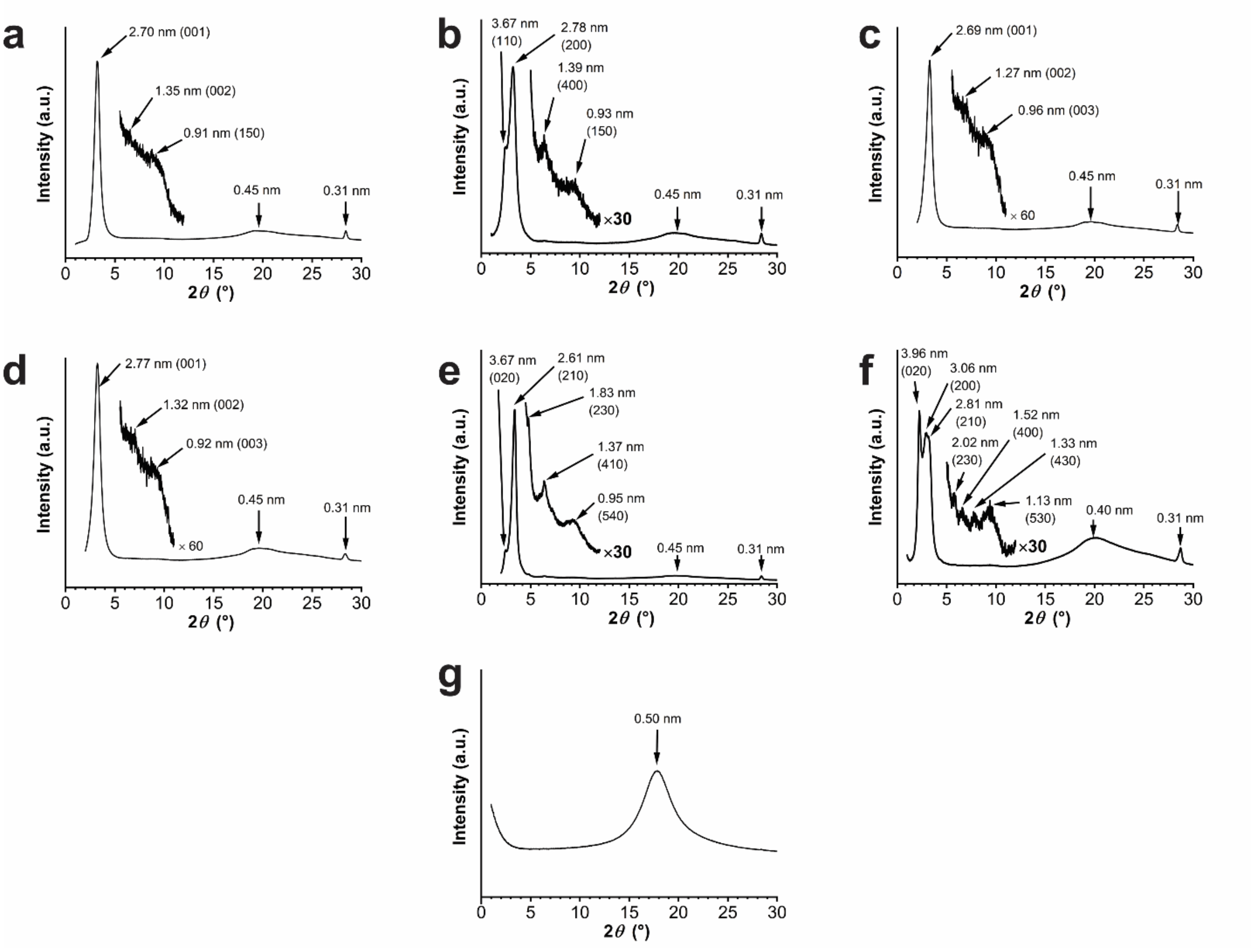

Figure S33. PXRD patterns of **ISA24**. (a) Xerogel (from cyclohexane gel, 1 wt %). (b) Xerogel (from cyclohexane gel, 1 wt %). (c) Xerogel (from chloroform gel, 1 wt %). (d) Xerogel (from toluene gel, 1 wt %). (e) Xerogel (from benzene gel, 2 wt %). (f) Aerogel (from benzene gel, 2 wt %). (g) Organogel with cyclohexane (2 wt %). All PXRD patterns were indexed using *LCDiXRay* [95] software.

Table S5. Powder X-ray diffraction data of lamello-columno phases of **ISA24** from dried gel samples.

**Sample Type**	**Solvent Type**	d_{obs} (Å)	**Peak shape**	***hkl***
Xerogel	Cyclohexane	2.68	s	*001*
		1.33	s	*002*
		0.94	w	*003*
		0.45	w, br	h_{ch}
		0.31	halo	h_0
Aerogel	Cyclohexane	2.69	s	*001*
		1.35	s	*002*
		0.94	w	*003*
		0.45	w, br	h_{ch}
		0.31	halo	h_0
Xerogel	Toluene	2.77	s	*001*
		1.32	s	*002*
		0.92	w	*003*
		0.45	w, br	h_{ch}
		0.31	halo	h_0
Xerogel	Chloroform	2.69	s	*001*
		1.27	s	*002*
		0.96	w	*003*
		0.45	w, br	h_{ch}
		0.31	halo	h_0
		0.31	m	h_0

s = strong, m = medium, w = weak, br = broad. All PXRD patterns were indexed using *LCDiXRay* [95] software.

Table S6. Powder X-ray diffraction data of rectangular columnar (Col_r) phases of **ISA24** from dried gel samples.

Sample Type	Solvent Type	d_{obs} (Å)	Peak shape	*hkl*	d_{calcd} (Å)	a_r (nm)	b_r (nm)
Xerogel	Cyclohexane	3.67	m	*110*	3.67*	5.50	4.93
		2.75	s	*200*	2.75*		
		1.39	w	*400*	1.38		
		0.93	w, br	*150*	0.97		
		0.45	halo	h_{ch}			
		0.31	m	h_0			
Aerogel	Cyclohexane	3.62	m	*110*	3.62*	5.42	4.86
		2.71	s	*200*	2.71*		
		1.38	w	*400*	1.36		
		0.98	w, br	*150*	0.96		
		0.44	halo	h_{ch}			
		0.32	m	h_0			
Xerogel	Benzene	3.59	s	*020*	3.59*	5.61	7.18
		2.61	s	*210*	2.61*		
		1.83	w	*230*	1.82		
		1.37	w	*410*	1.38		
		0.95	w, br	540	0.95		
		0.45	halo	h_{ch}			
		0.31	m	h_0			
Aerogel	Benzene	3.96	s	*020*	3.96*	6.12	7.92
		3.06	s	*200*	3.06*		
		2.81	s	*210*	2.85		
		2.02	w	*230*	2.00		
		1.52	w	*400*	1.53		
		1.33	w	*430*	1.32		
		1.13	w	*530*	1.11		
		0.95	w, br	*550*	0.97		
		0.45	halo	h_{ch}			
		0.31	m	h_0			

* chosen peaks for indexation. s = strong, m = medium, w = weak, br = broad. All PXRD patterns were indexed using *LCDiXRay* [95] software.

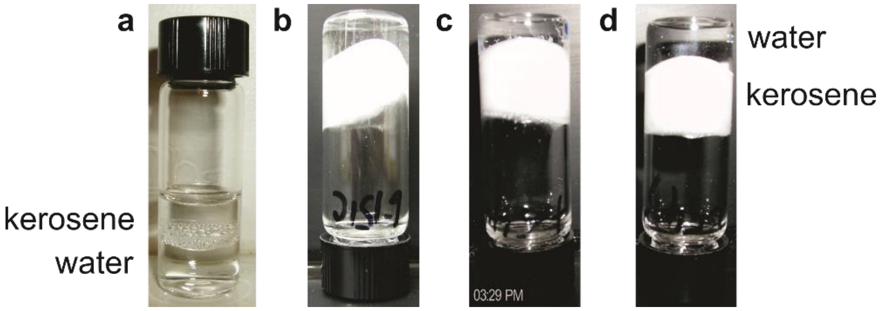

Figure S34. Digital photos of the phase selective gelation by **ISA24**. (a) Immiscible, 1:1 mixture of kerosene (1 mL) floating on top of water (1 mL). Inverted vials showing the phase selective gelation of kerosene by **ISA24** from 1:1 kerosene:water mixtures (2 mL) using the (b) the heating method (10.4 mg), and (c) the ultrasonic bath method (5 min., 9.6 mg/mL), and (d) the vortex mixing method (5 min., 10.4 mg/mL).
